# “Gold-Standard” Δ‑Machine Learned Transferable Potential for Linear Alkanes

**DOI:** 10.1021/acs.jpclett.5c02946

**Published:** 2025-11-20

**Authors:** Chen Qu, Apurba Nandi, Paul L. Houston, Qi Yu, Riccardo Conte, Joel M. Bowman

**Affiliations:** † Independent Researcher, Toronto, Ontario M9B0E3, Canada; ‡ Department of Physics and Materials Science, University of Luxembourg, L-1511, Luxembourg City, Luxembourg; § Department of Chemistry and Chemical Biology, Cornell University, Ithaca, New York 14853, United States; ∥ Department of Chemistry, 12478 Fudan University, Shanghai, 200438, P. R. China; ⊥ Dipartimento di Chimica, Università Degli Studi di Milano, via Golgi 19, 20133 Milano, Italy; # Department of Chemistry and Cherry L. Emerson Center for Scientific Computation, 1371 Emory University, Atlanta, Georgia 30322, United States

## Abstract

The conformational properties of linear alkanes, C_*n*_H_2*n*+2_, have been of intense interest for many years. Experiments and corresponding electronic structure calculations were first reported in the mid-2000s and continue to the present time. These focus on the minimum chain length where the transition from the linear minimum to the hairpin minimum occurs. We recently reported a transferable, many-body permutationally invariant polynomial (MB-PIP) potential for linear alkanes trained on roughly 253 000 B3LYP electronic energies for C_14_H_30_. Here we report a Δ-ML approach to elevate this B3LYP-based and a new PBE0+MBD MB-PIP potential using roughly 4500 Pair Natural Orbital Local Coupled Cluster (PNO-LCCSD­(T)-F12) energies. The new Δ-corrected potentials predict the difference in these minima accurately, compared to benchmark PNO-LCCSD­(T)-F12 energies results, over the range C_12_H_28_ to C_28_H_58_. Vibrational power spectra are also reported for C_14_H_30_ using the original B3LYP-based and Δ-ML corrected potentials. These new MB-PIP potentials for linear alkanes are the most accurate ones currently available and can be used in studies of properties of linear alkanes.

## Graphical abstract

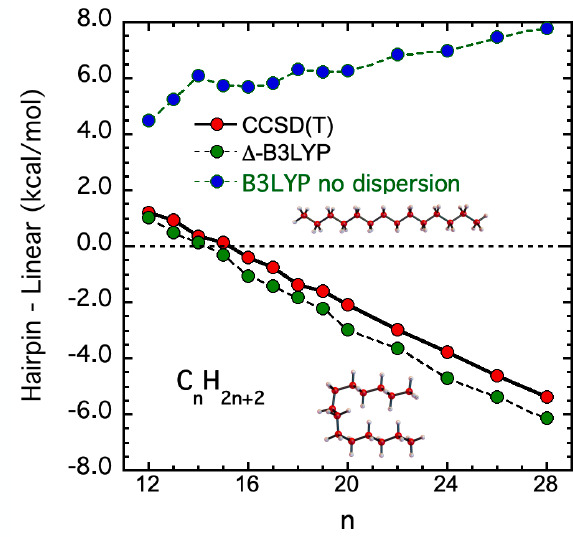

Hydrocarbons are important in fuels, plastics, and other industrial products. Consequently, there have been many studies, not only of their chemistry, but also of their physical and molecular properties. The conformational properties of linear alkanes, C_*n*_H_2*n*+2_, have been of particular interest for many years. Early studies of hydrocarbons have been reviewed by Patterson.^1^ Molecular mechanics models have been developed by Allinger,^2^ and by Jorgenson,^3–5^ among others.

Experiments and corresponding electronic structure calculations were reported in the mid-2000s. These studies focus on identifying the shortest chain length at which the hairpin structure becomes more stable, i.e., energetically more favorable, than the linear structure.^6–8^ (See Figure 1 for a depiction of the linear and hairpin conformers of alkanes, exemplified by C_14_H_30_.) Theoretical electronic structure work continues to the present. The paper by Liakos and Neese^8^ is a recent and comprehensive study of the linear and hairpin minima using high-level *ab initio* theory, namely Domain Based Pair Local Natural Orbital Coupled Cluster theory (DLPNO-CCSD­(T)). Rather than repeat the excellent background given there, we simply refer the interested reader to that paper. A major point of this careful study was the quantitative examination of the difference in geometry of these two minima and the resultant sensitivity of the energy difference. This was examined for alkanes ranging from C_6_H_14_ to C_18_H_38_, with the conclusion that the hairpin minimum drops below the “extended” linear one at C_16_H_34_ or C_17_H_36_. This conclusion is in accord with earlier ones using lower-levels of electronic structure theory.^6,7^ A final point about these “single-point” studies is that the importance of dispersion was noted by Bartlett and co-workers who wrote “As the length of unbranched alkane chains reaches some critical length, intramolecular dispersion forces cause a self-solvation effect in which the chains assume a folded conformation.”^7^ More recent, extended computational studies of linear alkane conformational energies by Ehlert et al.^9^ created the ACONFL data set of energies. Subsequent work using this data set was reported by Santra and Martin^10^ and most recently in 2023 by Werner and Hansen.^11^ The carefully benchmarked Pair Natural Orbital Local Coupled Cluster with Singles, Doubles, and Perturbative Triples with Explicit Correlation (PNO-LCCSD­(T)-F12) approach introduced by Werner and Hansen is used here (see below for details).

**Figure 1:**
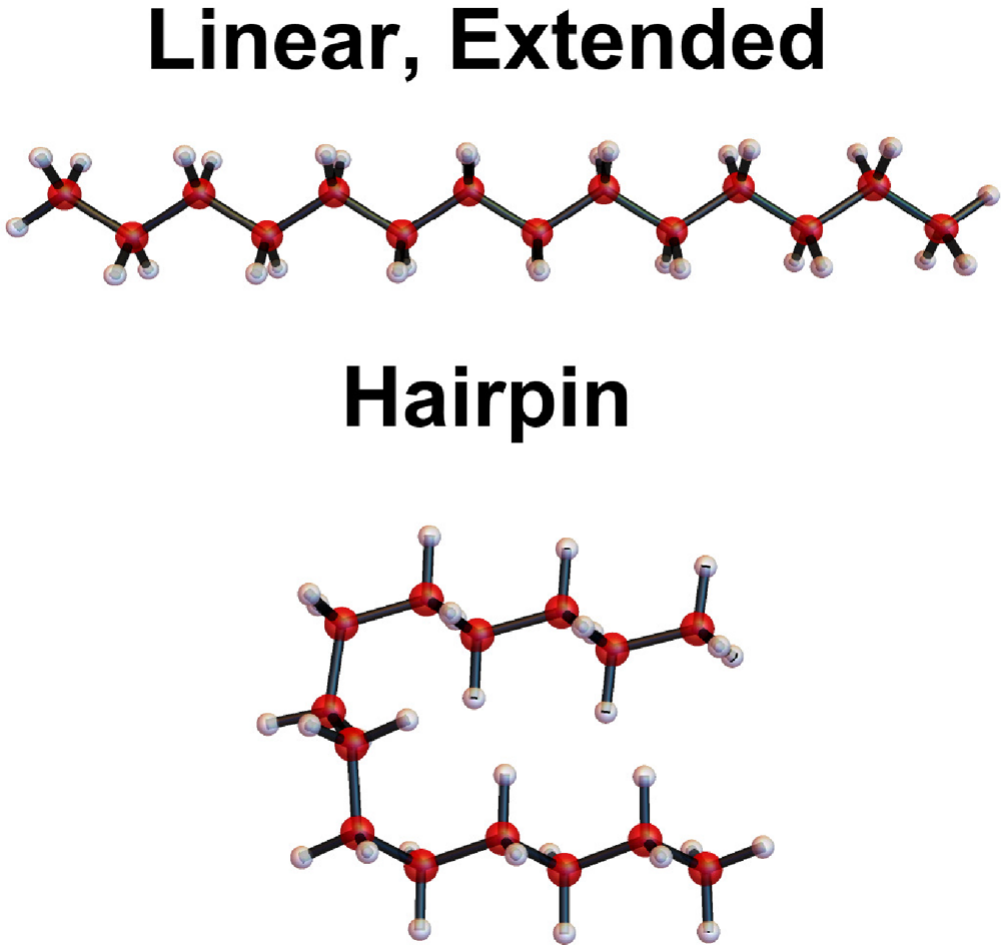
Linear extended and hairpin structures for the alkane C_14_H_30_, based on results in ref 12.

Since 2023, there has been substantial progress in methodology in developing machine-learned potentials; for a recent review see ref 13. With respect to linear alkanes, we recently reported a permutationally invariant polynomial (PIP), machine-learned potentials for C_14_H_30_ from fitting of roughly 253 000 B3LYP,^14,15^ with an aug-cc-pVDZ (aVDZ)­basis,^16^ electronic energies that span a large energy range up to 230 kcal/mol, relative to the global minimum. Details of this extensive data set are given in ref 12. The energies and forces were obtained using the Gaussian 16 computational chemistry package.^17^ More recently, we reported a novel many-body “targeted” approach, denoted MB-PIP, that we showed is transferable to general linear alkanes.^18^ This MB-PIP PES was recently used in a study of the energy landscape of folding in n-C_14_H_30_.^19^

Here, we report a Δ-machine learning (ML) approach to elevate the transferable B3LYP-based MB-PIP potential and a new PBE0+MBD potential fit to PBE0+MBD energies,^20–22^ computed with the “intermediate” basis settings of the FHI-aims electronic structure package,^23,24^ using PNO-LCCSD­(T)-F12b/AVTZ’ energies (where AVTZ’ means cc-pVTZ basis for H and aug-cc-pVTZ for C).^11^ This is a major advance in applying the Δ-ML approach to a transferable potential for alkanes.

The paper is organized as follows. First, we give a brief review of many-body approaches in machine-learned potentials, including those based on PIP regression. Then, details of the approach taken for the linear alkanes are reviewed followed by details of the Δ-ML approach. The results of the approach are given and assessed by comparing the energy difference between the hairpin and linear minima against the benchmark numbers. Power spectra of C_14_H_30_ are presented using B3LYP and Δ-B3LYP potentials at two total energies corresponding to temperatures of 10 and 300 K.

Many-body approaches to machine learned potentials of large molecules, clusters, and condensed phases are becoming the standard approach; however, the details vary greatly depending on the method. For a recent review including a list of more than 90 open-source software programs, see ref 13. Among this large group of approaches, permutationally invariant polynomial regression has played an important role, both historically and also very recently.^25^ This is reviewed briefly below which concludes with the details of a new Δ-machine learning approach for our MB-PIP potentials. That approach is applied to two DFT-based MB-PIP potentials for linear alkanes.^12,18,26^

Many-body representations of potentials describing noncovalent interactions are generically given by

1 $$V\left(1,...,N\right)=\overset{N}{\underset{i=1}{\sum }}V_{1-b}\left(i\right)+\overset{N}{\underset{i>j}{\sum }}V_{2-b}\left(i,j\right)+\overset{N}{\underset{i>j>k}{\sum }}V_{3-b}\left(i,j,k\right)+\overset{N}{\underset{i>j>k>l}{\sum }}V_{4-b}\left(i,j,k,l\right)+\cdot \cdot \cdot$$

where *N* is the number of “bodies” e.g., for water each “body” is a water monomer.^27–31^ In these widely known potentials each *n*-body interaction is fit to “gold-standard” CCSD­(T) energies using a basis of PIPs. The most recent of these, q-AQUA,^29^ q-AQUA-pol,^30^ and MB-pol(2023)^31^ fit up to 4-body interactions. Details about each term in the above equation for the water potential are given in those papers.

For covalent interactions, i.e., for a single (large) molecule, MB-PIP approaches are just appearing in the literature. In this case, there is not an obvious partitioning in terms of “bodies”. Paesani and co-workers recently suggested a physically based partitioning of linear hydrocarbons and developed a transferable PIP-based model.^32^ Shortly after, we proposed and tested an atom-based PIP-approach that is different from – albeit inspired by – a PIP representation of an atom-centered expansion approach exemplified by work of Csanyi and co-workers.^33^ In our approach^12,26^ the monomers are atoms, and these interactions are enumerated for all atoms in the molecule. For an *n*-body interaction among *N* atoms, there are a total of *N*!/[*n*!(*N* – *n*)!] interactions. To limit the number of interactions, physical range cutoffs are used for each *n*-body interaction, and details can be found in refs 12, 18 which report our first application to the 44-atom alkane C_14_H_30_ and transferability to alkanes as large as C_30_H_62_, respectively. In this example (and also for other two-element molecules, clusters, materials, etc.,) there are three types of 2-b interactions, i.e., CC, HH, and CH interactions, four types of 3-b, and five types of 4-b interactions. (We do not consider 5-b interactions.) For each interaction beyond 2-b, an appropriate PIP basis is used and there are fewer of these than the types of interactions. To see this, consider the 3-b CCC and HHH interactions. While these are different, the PIP basis is the same, namely the basis for *A*_3_ but the corresponding sets of linear coefficients are different. The full expression, up to *V*_4–*b*_, for this MB-PIP representation has been given previously;^18^ here we just illustrate this for *V*_3–*b*_.

2 $$V_{3-b}=\underset{C_{i},C_{j},C_{k}}{\sum }V_{3-b}^{\left(\mathrm{CCC}\right)}\left(y_{ij},y_{ik},y_{jk}\right)+\underset{C_{i},C_{j},H_{k}}{\sum }V_{3-b}^{\left(\mathrm{CCH}\right)}\left(y_{ij},y_{ik},y_{jk}\right)+\underset{C_{i},H_{j},H_{k}}{\sum }V_{3-b}^{\left(\mathrm{CHH}\right)}\left(y_{ij},y_{ik},y_{jk}\right)+\underset{H_{i},H_{j},H_{k}}{\sum }V_{3-b}^{\left(\mathrm{HHH}\right)}\left(y_{ij},y_{ik},y_{jk}\right)=\underset{m}{\sum }\underset{C_{i},C_{j},C_{k}}{\sum }c_{m}^{\left(\mathrm{CCC}\right)}p_{m}^{\left(A_{3}\right)}\left(y_{ij},y_{ik},y_{jk}\right)+\underset{m}{\sum }\underset{C_{i},C_{j},H_{k}}{\sum }c_{m}^{\left(\mathrm{CCH}\right)}p_{m}^{\left(A_{2}B\right)}\left(y_{ij},y_{ik},y_{jk}\right)+\underset{m}{\sum }\underset{C_{i},H_{j},H_{k}}{\sum }c_{m}^{\left(\mathrm{CHH}\right)}p_{m}^{\left(A_{2}B\right)}\left(y_{jk},y_{ij},y_{ik}\right)+\underset{m}{\sum }\underset{H_{i},H_{j},H_{k}}{\sum }c_{m}^{\left(\mathrm{HHH}\right)}p_{m}^{\left(A_{3}\right)}\left(y_{ij},y_{ik},y_{jk}\right)$$

where the first term represents the interaction of three C atoms, the second the interaction of two C and one H atom, etc. The corresponding permutational symmetry of these two 3-b PIPs are *A*_3_ and *A*_2_ *B*, etc., and *y*_*ij*_ are transformed internuclear distances (such as Morse variables *y*_*ij*_ = exp­(−*r*_*ij*_/λ), where λ is a range (hyper)­parameter).

For the expansion up to 4-b terms, the general permutational symmetry of the PIPs bases are *A*_4_, *A*_3_ *B*, and *A*_2_ *B*_2_. Also, we note that the distinct 2-b, 3-b, and 4-b PIP bases can have different range parameters for the corresponding Morse variables, and different maximum polynomial orders. Once these bases are set up, the linear coefficients are determined using a standard overdetermined linear least-squares fit to the total energies of this molecule. Note, the MB-PIP approach with up to 4-body terms involves diatomic, triatomic, and tetraatomic “molecules”, a variety of permutationally invariant methods such as PIP-NN/FI-NN^34,35^ and PIP-GPR/FI-GPR^36^ can also be employed.

As shown previously, this MB-PIP is transferable to linear alkanes and this was demonstrated for alkanes with as many as 30 carbon atoms.^37^ We also note that the MB-PIP approach is to be distinguished from fragmentation representations^38^ and the use of a fragmented PIP basis.^39^ This approach was used successfully to represent the PES for C_14_H_30_.^12^

This MB-PIP potential was fit to a data set of roughly 253 000 B3LYP energies for C_14_H_30_; details of the data set and fitting are given elsewhere,^12,18^ and briefly summarized below. We note here and also in a very recent paper^19^ that dispersion is not accounted for in these straight B3LYP energies. Here the goal is to correct this and other deficiencies of this functional by using Δ-ML at the CCSD­(T) level. In addition, the above expression for the MB-PIP is employed here for a new set of PBE0+MBD energies,^20–22^ computed with the “intermediate” basis settings of the FHI-aims electronic structure package,^23,24^ for the same set of configurations. Since this approach does contain dispersion, Δ-ML applied to this PES is not as heavy a “lift”. In general, Δ-ML at the CCSD­(T) level can correct errors in DFT that include lack of or inaccurate treatments of dispersion, limitations in the basis set and description of exchange-correlation

The Δ-ML approach is given by the general equation^40,41^

3 $$V_{LL\to CC}=V_{LL}+\Delta V_{CC-LL}$$

where *V*_*LL*→*CC*_ is the corrected PES, *V*_*LL*_ is a PES fit to low-level DFT electronic data, and Δ*V*_*CC*–*LL*_ is the correction PES based on high-level (usually coupled cluster) energies. We have introduced this approach for MLPs for larger molecules such as acetylacetone,^42^ tropolone,^43^ and most recently to ethanol.^44^ In this application, we applied the procedure successfully to several DFT functionals. We have also extended this approach to noncovalent interactions, specifically water, where the low-level potential was actually a sophisticated forced field.^30^ In the present context *V*_*LL*_ is the above MB-PIP fit to B3LYP or PBE0+MBD energies and Δ*V*_*CC*–*LL*_ is the analogous fit to the difference between high-level and DFT energies. Here we apply PNO-LCCSD­(T)-F12 energies, implemented in Molpro,^45^ to obtain the correction term Δ*V*_*CC*–*LL*_. Details of the MB-PIP fits are given in the Supporting Information (SI).

From these parameters there are 734 linear coefficients to be determined for the entire MB-PIP expression. These were done in a single least-squares fit to *ab initio* energies of C_14_H_30_. (See ref 46 for details of the least-squares implementation.) Training on C_14_H_30_ gives a root-mean-square error on energies of 115 cm^–1^ (0.33 kcal/mol), and corresponds to an error of 2.6 cm^–1^ (0.007 kcal/mol) per atom. This is a very small precision error, compared to typical ML values for such a large molecule and data set. Even though it should be clear, *prima facie*, these many-body terms, trained on C_14_H_30_, are transferable to all alkanes. The precision of such transferability needs to be verified of course. This was done by us previously for the B3LYP-based PES, where excellent precision was shown over the range C_4_H_10_ to C_30_H_62_.^18^

For Δ*V*_*CC*–*LL*_, a corresponding MB-PIP fit was done to 4514 energy differences between PNO-LCCSD­(T)-F12b and B3LYP or PBE0+MBD ones for C_14_H_30_. The 4514 configurations were chosen as follows: A first set of 1359 configurations comes from molecular dynamics simulations. These calculations were done with our own Fortran codes, developed for many applications and for over more than two decades. Specifically, along the minimum energy path between the linear and hairpin structure, 7 additional local minima were located on the low-level PES. Molecular dynamics calculations (NVE) starting from these 9 stationary points (7 plus the linear and hairpin) were carried out and 151 configurations were collected from each trajectory. The second set of 459 configurations was obtained by assigning random displacement of coordinates to the 9 stationary points, 51 configurations each. The last 2544 configurations were randomly picked from the data set used for the low-level PES. For all the 4514 configurations, PNO-LCCSD­(T)-F12 energies were obtained. A histogram of the energy distribution of this coupled-cluster data set is shown in Figure 2. The fitting of *V*_*LL*→*CC*_ also used the MB-PIP approach, with the same hyperparameters as the low-level PES, except the polynomial order. The maximum polynomial orders of 2-b, 3-b, and 4-b of the Δ*V*_*CC*–*LL*_ are 6, 7, and 5, respectively, resulting in 383 unknown coefficients. This very small number results in a trivial linear least-squares problem. The fitting errors are 12 cm^–1^ for the Δ-B3LYP data and 7 cm^–1^ for the Δ-PBE0+MBD data; these indicate very precise fitting. These small fitting errors are actually not surprising and do not indicate overfitting. This is because the range of the energy difference is much smaller, specifically, the energy differences between PNO-LCCSD­(T)-F12 and B3LYP span a range of 12 966 cm^–1^, and the differences between PNO-LCCSD­(T)-F12 and PBE0+MBD span only 3904 cm^–1^. We note that the larger range for the former than the latter is not surprising, because the B3LYP energies are dispersionless whereas the PBE0+MBD ones describe dispersion accurately. That the precision of the two correction PESs, Δ*V*_*CC*–*LL*_, are about the same is certainly a gratifying outcome of our machine learning approach.

**Figure 2:**
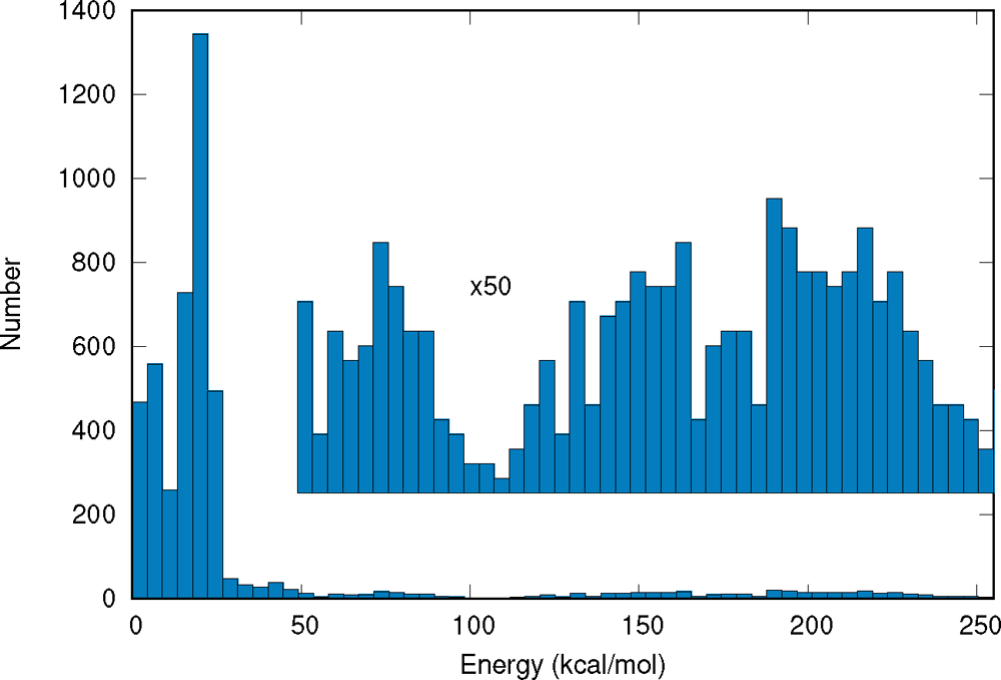
Energy distribution of the coupled-cluster data set used for fitting the Δ corrections.

To sum up thus far, we have obtained transferable low-level MB-PIP PESs based on B3LYP (with no dispersion) and PBE0+MBD energies, *V*_*LL*_, and corresponding correction PESs Δ*V*_*CC*–*LL*_, all trained on C_14_H_30_. We now examine the performance of the transferability of the corrected PES, *V*_*LL*→*CC*_ for the determination of the energy difference between the hairpin and extended conformations over the range C_12_H_26_ to C_28_H_58_. For consistency, we use stationary configurations for these alkanes, obtained from direct optimization of the relatively efficient and (as seen below) accurate PBE0+MBD method.

To begin we show results for the B3LYP and the B3LYP-Δ-corrected PES, denoted Δ-B3LYP, graphically in Figure 3. To be clear the “B3LYP” results are from the transferable MB-PIP B3LYP-PES (*V*_*LL*_) and those labeled Δ-B3LYP are from the transferable MB-PIP corrected Δ-B3LYP PES (*V*_*LL*→*CC*_). Also, to be clear “PES” refers to the MB-PIP machine-learned potential. As seen, there is a major improvement of the B3LYP PES (with no dispersion) such that the corrected PES is very close to the benchmark CCSD­(T) results. The correction is not perfect, as expected, with a gap of roughly 0.5 kcal/mol. This can be traced mainly to the fact that Δ*V*_*CC*–*LL*_ was trained on the difference between direct B3LYP and CCSD­(T) energies and not the difference between the B3LYP PES and CCSD­(T) energies. Across the range of alkanes shown the mean absolute error of the PES is 0.5 kcal/mol. We note that the importance of dispersion in alkane conformation has been known for years^6–9,47,48^ and so we are not adding to that knowledge. However, the results in Figure 3 provide quantitative measure of dispersion over this large range of alkanes.

**Figure 3:**
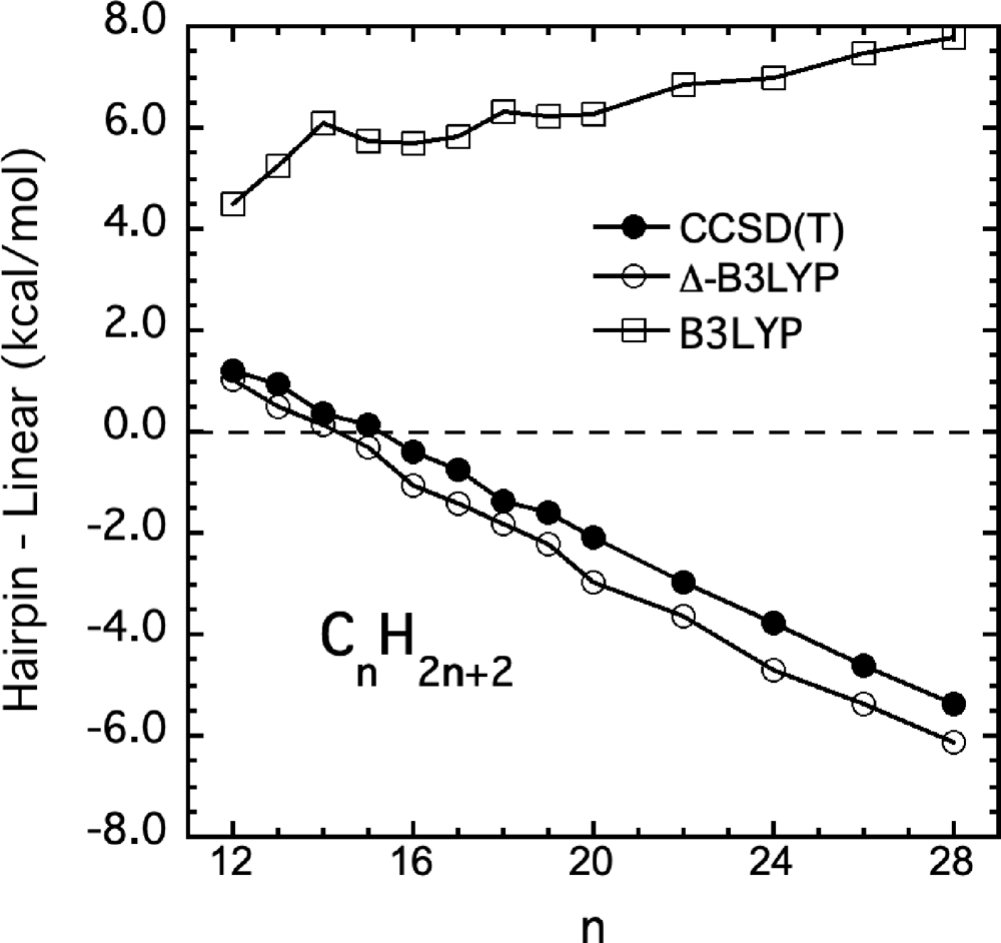
Energy difference between hairpin and linear minima from the original B3LYP-based MB-PIP transferable potential,^18^ the present Δ-ML corrected one, denoted Δ-B3LYP, and “CCSD­(T)”, which refers to PNO-LCCSD­(T)-F12.

Next, consider the performance of the PBE0+MBD MB-PIP and Δ-corrected PBE0+MBD PESs for these energy differences. These are shown in Figure 4. As seen the PBE0+MBD results are in good agreement with the CCSD­(T), with differences of roughly 1–1.5 kcal/mol. The corrected PBE0+MBD PES differences are reduced substantially to about 0.5 kcal/mol; essentially the same difference as seen for the corrected B3LYP MB-PIP PES. The numerical values shown in Figures 3 and 4 are given in Table 1 in the . These results indicate that the new transferable corrected MB-PESs based on B3LYP and PBE+MBD have successfully been elevated to the “gold-standard” level, especially the previous B3LYP one.

**Figure 4:**
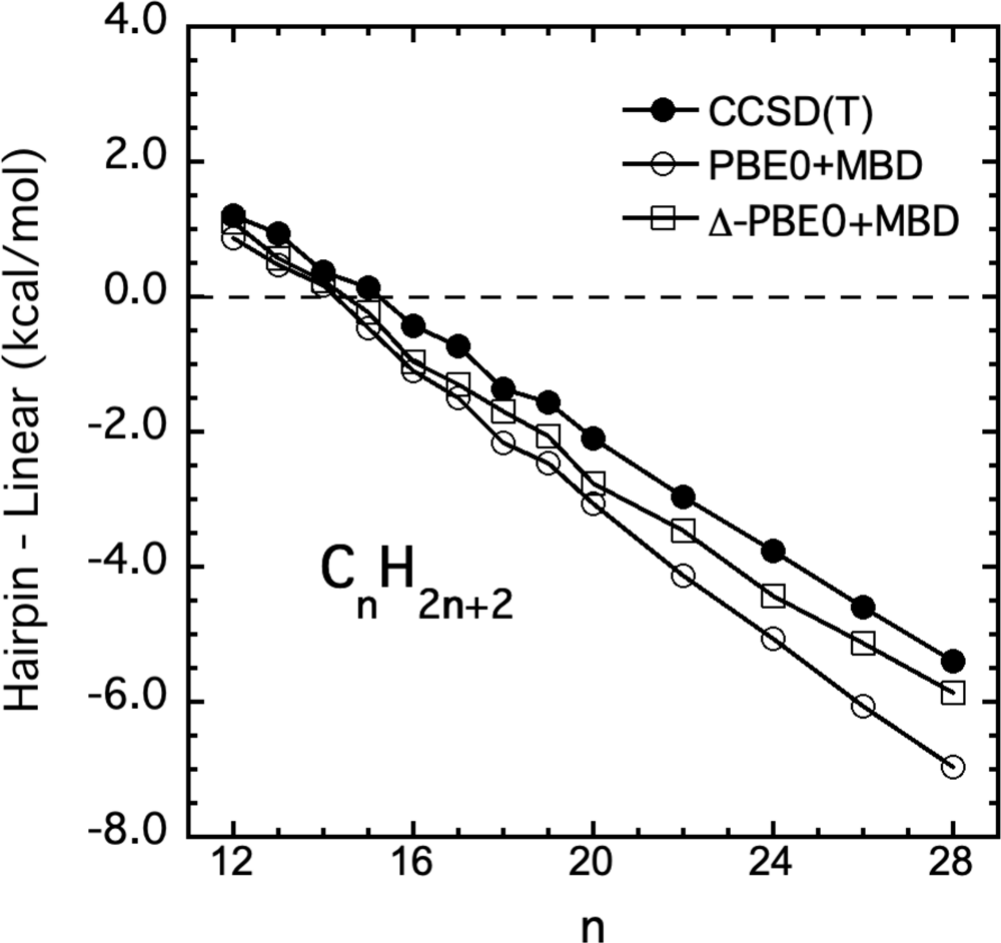
Energy difference between hairpin and linear minima from the PBE0+MBD-based MB-PIP transferable potential, the Δ-ML corrected one, denoted Δ-PBE0+MBD, and “CCSD­(T)”, which refers to PNO-LCCSD­(T)-F12.

There are numerous studies that can be done with these corrected MB PESs. (And we note all the MB-PIP PESs have fast analytical gradients, i.e., the cost of the gradient, obtained via “reverse differentiation,” is roughly three times the cost of the energy evaluation; see refs 49 and 50 for details.) One that we present here are calculations of the vibrational power spectra of C_14_H_30_ from molecular dynamics simulations, using the B3LYP PES and Δ-B3LYP PES. These were performed as we did recently,^26^ by running a classical trajectory initiated from a specific configuration. Specifically, the classical trajectories adopted a step size of 5.0 au (0.121 fs); each trajectory were equilibrated for 10,000 steps (1.2 ps), and continued for another 22,500 steps (2.7 ps). The final spectra are averages for five trajectories at each temperature and potential. Here we considered both the linear and hairpin minima. The trajectories were run using the NVE protocol and for zero total angular momentum using our in-house software. The total energy is obtained from the simple correspondence between the average energy ⟨*E*⟩ and temperature *T* for classical harmonic oscillators. (For C_14_H_30_ ⟨*E*⟩ = 126*RT*.) Temperatures of 10 and 300 K were considered with the goal of examining both the difference in the spectra at these two temperatures and also the differences between the B3LYP PES and Δ-B3LYP PES. The power spectra were obtained by using an open source web platform,^51,52^ recently developed at the University of Milan, which is based on the time-averaged Fourier transform of the Cartesian velocity autocorrelation function.^53^ These spectra are shown in Figure 5.

We examined such trajectories in detail previously,^26^ and noted that at 300 K many conformations are visited and so the final spectra are virtually identical whether starting from the hairpin or linear minimum. However, at 10 K this is not the case, and the spectra obtained are more representative of the harmonic normal mode spectra at these minima. With this in mind consider the 10 K results first. There are clear differences between the hairpin and linear spectra in the range 0 to 1000 cm^–1^ and this is seen for both the B3LYP and Δ-B3LYP potentials. And, not surprisingly, we also see significant differences between these potentials in this spectral range. These differences are largely quenched for the 300 K spectra for both potentials. So, one conclusion from these results is that signatures of the effects of dispersion are present at 10 K in the spectral range 0 to 1000 cm^–1^. This conclusion is consistent with the double harmonic analysis done by Luttschwager and Suhm in 2013.^6^

A second, and again not surprising, conclusion from these spectra is that the high-frequency CH-stretch region is largely insensitive to temperature and potential.

The present results demonstrate that it is straightforward to elevate a low-level, e.g., DFT, MB-PIP potential for a large class of molecules, e.g., linear alkanes, to the CCSD­(T) level. This was done here for MB-PIP architecture, where all terms are fit “at once”. That the method is successful in elevating a low-level PES based on dispersion-less B3LYP calculations is very encouraging, albeit not necessarily surprising. Note the correction to the low-level PES was done by fitting a difference potential using the “at once” approach. The high-level energies were obtained for C_14_H_30_ using efficient and certified PNO-LCCSD­(T)-F12 methods in Molpro 2023. Also, we note that correction terms could be obtained using a smaller alkane. This is something to be investigated in the future.

The success was explicitly demonstrated for the energy difference between the hairpin and linear conformers of alkanes ranging from 12 to 28 carbon atoms against CCSD­(T) energies. As noted above the most recent “single-point” study of alkanes considered data sets of 13, 18, and 22 configurations for alkanes with 12, 16, and 20 carbon atoms.^9^ The present work considered 4514 configurations, spanning a large energy range, at which PNO-LCCSD­(T)-F12 energies were obtained. These were used to precisely fit a MB-PIP difference potential with precision of less than 0.03 kcal/mol.

Finally, we note a different, “building block”, approach was recently introduced by Paesani and co-workers for polyalanine chains.^54^ This work uses and builds on the many-body approach based on the selection of monomers, “chemically intuitive building blocks.”^32^ In the more recent paper on polyalanine, each building block is separately elevated to the CCSD­(T) level (using the DLPNO–CCSD­(T) method). This is certainly a reasonable approach. However, a statement made in that paper about our MB-PIP approach is contradicted by the present results. Specifically, it was stated that “···the ‘many-body strategy’ builds PIP terms for interactions involving sets of 2, 3, or 4 atoms, which are then summed to predict the total molecular energy··· because these models require whole-molecule reference data for training, they are typically limited to DFT-level calculations due to the prohibitive cost of higher-level quantum methods.” The present work demonstrates that the MB-PIP approach can extend to higher-level methods using a standard Δ-ML approach and without the need to select molecule-specific building blocks. It should also be noted that recently, a conceptually different approach, MB-PIPNet, was introduced by some of us, which decomposes the total energy of the system into sum of effective monomeric energies using only monomeric 1-b and 2-b descriptors.^55^ Extension of the MB-PIPNet framework to systems such as alkanes is under investigation and will be reported in the future.

To summarize, the present paper reports two significant results. One is the successful elevation of DFT-based, transferable MB-PIP potentials for alkanes to the CCSD­(T) level. Second, we demonstrated quantitatively the major and increasing importance of dispersion in determining the folding conformation of linear alkanes.

This new transferable potential can now be used in many applications and tests of other potentials for this important class of molecules. For example, our most recent work was a study of energies and configurations of tens of thousands of local minima and first order saddle-points of C_14_H_30_ using the B3LYP MB-PIP PES.^19^ It is now possible to conduct that study for a MB-PIP PES that includes dispersion and also for a range of alkanes. Also, the new MB-PIP PES can be used to test the new generation of universal machine-learned ones, such as MACE-OFF,^56^ SO3LR,^57^ ANI,^58^ Allegro,^59^ and OMNI-P1.^60^ Our expectation is that these methods will run slower than the current one owing to the much larger number of learned parameters and complexity of these approaches. We have tested the timings on one of these approaches, specifically, MACE-OFF. It takes 56.7 s to compute the energies and gradients of 500 configurations on an Intel Xeon Gold 6542Y CPU, and 11.0 s on the NVIDIA RTX 4000 Ada GPU (using CUDA acceleration with the cuEquivariance library). The Δ-corrected MB-PIP approach takes only 2.49 s for the same number of configurations on the same CPU. Finally, other dispersion corrections to DFT can be tested against the current CCSD­(T) results for the minima and also the data set of 4514 energies, which are available, see below.

**Figure 5:**
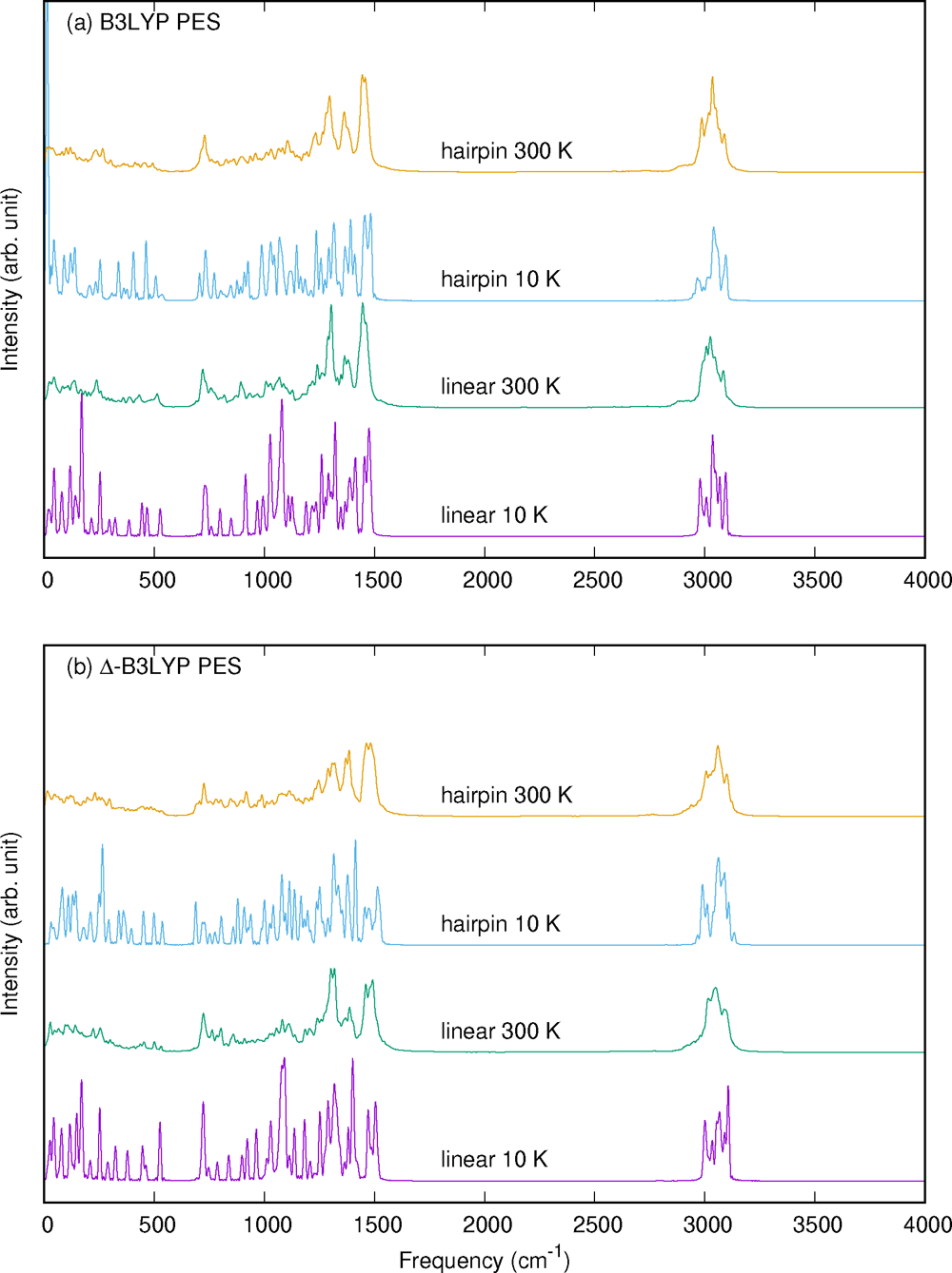
Power spectra of C_14_H_30_ using B3LYP and Δ-B3LYP PES at indicated temperatures.

## Supplementary Material





## Data Availability

The PNO-LCCSD­(T)-F12 electronic energies used in this paper are available at https://github.com/jmbowma/QM-22, and the MB-PIP PES is available upon request to the authors.

## Notes

Details of the MB-PIP fits are given there, along with a table containing the energy differences of all the mininma shown in figures. The Cartesian coordinates of all linear and hairpin minima obtained from direct PBE0+MBD optimization are also given. Commonly reported energy units are used in this paper. These may be converted to SI units using the equivalences 4.184 kJ/mol = 1 kcal/mol = 350 cm^–1^ = 43 meV (PDF)
Transparent Peer Review report available (PDF)
