# Supplementary material for: “Gold-Standard” Δ‑Machine Learned Transferable Potential for Linear Alkanes
Source: J Phys Chem Lett. 2025 Nov 20;16(48):12393–400. doi: 10.1021/acs.jpclett.5c02946 (PMC12683630; doi:10.1021/acs.jpclett.5c02946)
Supplement: Supplementary file 1 [file jz5c02946_si_001.pdf]

Supporting Information "Gold-Standard" Δ-Machine Learned and Transferable Potential for Linear Alkanes

Chen Qu,[*,†] Apurba Nandi,[*,‡] Paul L. Houston,[§] Qi Yu,[‖] Riccardo Conte,[⊥] and Joel M. Bowman[*,#]

†*Independent Researcher, Toronto, Ontario M9B0E3, Canada*

‡*Department of Physics and Materials Science, University of Luxembourg, L-1511, Luxembourg City, Luxembourg.*

¶*Dipartimento di Chimica, Università degli Studi di Milano, via Golgi 19, 20133 Milano, Italy*

§*Department of Chemistry and Chemical Biology, Cornell University, Ithaca, New York 14853, USA and Department of Chemistry and Biochemistry, Georgia Institute of Technology, Atlanta, Georgia 30332, USA*

‖*Department of Chemistry, Fudan University, Shanghai, 200438, P. R. China*

⊥*Dipartimento di Chimica, Università Degli Studi di Milano, via Golgi 19, 20133 Milano, Italy*

#*Department of Chemistry and Cherry L. Emerson Center for Scientific Computation, Emory University, Atlanta, Georgia 30322, USA.*

E-mail: szquchen@gmail.com; apurba.nandi@uni.lu; jmbowma@emory.edu

Details of the Fits

The hyperparameters used in the V_{LL} are as follows: In general, the expectation is that the 4-b interactions are shorter-ranged than 3-b than 2-b, so we applied smaller Morse range parameter and physical cutoff distance for 4-b, and larger values for 2-b. The 1-b energy is simply the sum of the energies of isolated carbon and hydrogen atoms computed using B3LYP/cc-pVDZ theory and it is not a trainable parameter. For the 2-b, a single Morse range parameter of 2.5 bohr was used, with a maximum power of 10, so the total number of 2-b coefficients is 30. The cutoff distance for 2-b is 18.0 bohr. For the 3-b, the Morse range parameter is 1.8 bohr, and the maximum polynomial order of the PIPs is 8. The switching function is applied when the maximum distance in a trimer is between 12.3 and 14.2 bohr, and the energy contribution is 0 when the maximum internuclear distance in the trimer is beyond 14.2 bohr. For the 4-b bases, the Morse range parameter is 1.2 bohr, and the maximum polynomial order is 6. The switching range for 4-body is between 8.5 and 10.4 bohr. All the bases are purified,[1,2] that is, any polynomial that does not go to zero when an atom is infinitely far away from the remaining atoms is removed from the bases. Using the polynomial orders mentioned above, there are 32, 78, 78, 32, 40, 115, 174, 115, and 40 PIPs for CCC, CCH, CHH, HHH, CCCC, CCCH, CCHH, CHHH, HHHH, respectively. These sum up to 734 undetermined linear coefficients, including the 30 from all the 2-b interactions.

Table of energy differences (kcal/mol) between hairpin and linear minima

Table 1: Energy difference (kcal/mol) between hairpin and linear minima from indicated calculations, and where "CCSD(T)" refers to direct PNO-LCCSD(T)-F12 calculations, and all other values were obtained using the corresponding MB-PIP PES. These are all evaluated at the PBE0+MBD optimized configurations.

n	CCSD(T)	B3LYP	Δ-B3LYP	PBE0+MBD	Δ-PBE0+MBD
12	1.20	4.50	1.00	0.92	1.10
13	0.92	5.24	0.50	0.46	0.58
14	0.37	6.07	0.14	-0.10	0.22
15	0.12	5.75	-0.33	-0.52	-0.25
16	-0.42	5.69	-1.08	-1.24	-0.98
17	-0.75	5.82	-1.42	-1.67	-1.29
18	-1.36	6.30	-1.83	-2.29	-1.71
19	-1.58	6.23	-2.20	-2.67	-2.06
20	-2.09	6.25	-2.96	-3.34	-2.77
22	-2.98	6.85	-3.65	-4.26	-3.45
24	-3.77	6.96	-4.70	-5.31	-4.43
26	-4.61	7.45	-5.39	-6.07	-5.12
28	-5.39	7.78	-6.15	-6.88	-5.86

PBE0+MBD Optimized Geometries of Hydrocarbons

$C_{12}H_{26}$ Linear Conformer				$C_{12}H_{26}$ Hairpin Conformer			
38				38			
-12859.639694955 eV				-12859.602146572 eV			
C	-0.06034529	0.04620656	-0.00000008	C	-0.04382949	0.01193077	0.24263735
C	1.45732338	0.02025666	0.00000003	C	1.47324383	0.01521080	0.18904916
C	2.02554666	-1.38842225	0.00000008	C	2.06450342	-1.38254348	0.12606178
C	3.54327671	-1.43174241	0.00000000	C	3.58273680	-1.39073466	0.05711164
C	4.10514933	-2.84255354	0.00000001	C	4.20032372	-2.78373970	-0.01233023
C	5.62279873	-2.89090408	-0.00000003	C	3.75648393	-3.62011378	-1.20683414
C	6.18184620	-4.30289654	-0.00000001	C	4.16353017	-3.04154900	-2.55348457
C	7.69951063	-4.35146723	0.00000002	C	3.55194836	-3.76311322	-3.74880029
C	8.26117190	-5.76244210	-0.00000001	C	2.03076876	-3.68585762	-3.83186852
C	9.77896473	-5.80597794	-0.00000001	C	1.48010510	-2.27163064	-3.91388279
C	10.34713968	-7.21471488	0.00000002	C	-0.03611401	-2.22048911	-3.98934850
C	11.86486195	-7.24090686	-0.00000001	C	-0.57612408	-0.80328687	-4.05593129
H	-0.44673237	1.06696003	-0.00000002	H	-0.44928405	1.02483961	0.27441217
H	-0.46140987	-0.46105743	0.88088306	H	-0.40422488	-0.51815010	1.12761681
H	-0.46140975	-0.46105726	-0.88088336	H	-0.46643265	-0.48789593	-0.63281774
H	1.83523469	0.56036584	-0.87479640	H	1.80938721	0.58450172	-0.68477382
H	1.83523455	0.56036583	0.87479653	H	1.87347956	0.54033233	1.06296540
H	1.64740122	-1.93004349	-0.87528779	H	1.64655334	-1.89707402	-0.74540456
H	1.64740130	-1.93004341	0.87528802	H	1.73634092	-1.95780803	1.00059469
H	3.92157642	-0.89072888	0.87545885	H	3.98060505	-0.87424717	0.93707690
H	3.92157635	-0.89072886	-0.87545887	H	3.90479172	-0.79547784	-0.80390432
H	3.72573941	-3.38294214	-0.87533278	H	3.95261127	-3.32416271	0.90772394
H	3.72573948	-3.38294207	0.87533288	H	5.29151130	-2.68806109	-0.02486536
H	6.00266203	-2.35092049	0.87539822	H	2.67138519	-3.74685675	-1.16922127
H	6.00266198	-2.35092051	-0.87539831	H	4.17451122	-4.62797923	-1.11343927
H	5.80192763	-4.84283596	-0.87539838	H	5.25505535	-3.07193238	-2.63680660
H	5.80192764	-4.84283593	0.87539837	H	3.89407443	-1.98318653	-2.60042242
H	8.07903614	-3.81116535	0.87534046	H	3.85331008	-4.81607103	-3.72688869
H	8.07903618	-3.81116533	-0.87534039	H	3.97569822	-3.34419042	-4.66795948
H	7.88278483	-6.30338525	-0.87546495	H	1.58281170	-4.19466125	-2.97175016
H	7.88278483	-6.30338527	0.87546492	H	1.69871463	-4.24749458	-4.71146087
H	10.15716715	-5.26442063	0.87529894	H	1.90687675	-1.76459956	-4.78805635
H	10.15716715	-5.26442068	-0.87529898	H	1.80389860	-1.69195573	-3.04325026
H	9.96929619	-7.75486004	-0.87480302	H	-0.45424830	-2.73281268	-3.11566310
H	9.96929619	-7.75486005	0.87480304	H	-0.37615222	-2.78758009	-4.86253332
H	12.25089700	-8.26181379	-0.00000003	H	-1.66679559	-0.78811636	-4.09633033
H	12.26613553	-6.73383314	0.88089185	H	-0.20356897	-0.28228653	-4.94127030
H	12.26613549	-6.73383313	-0.88089188	H	-0.26789140	-0.22420917	-3.18159327

$C_{13}H_{28}$ Linear Conformer				$C_{13}H_{28}$ Hairpin Conformer			
41				41			
-13928.573224782 eV				-13928.552492808 eV			
C	-0.06707852	0.05061651	0.00000004	C	0.11416290	0.30933330	0.01889073
C	1.45057157	0.02445645	-0.00000002	C	1.62064563	0.15398344	-0.07957751
C	2.01865183	-1.38428430	-0.00000002	C	2.07680579	-1.29217897	0.00889150
C	3.53635078	-1.42751641	0.00000000	C	3.58516946	-1.45196448	-0.05564887
C	4.09887878	-2.83810501	0.00000001	C	4.04002313	-2.89719413	0.06594747
C	5.61656365	-2.88471059	-0.00000002	C	5.55399478	-3.08517145	0.05571928
C	6.17829865	-4.29558621	-0.00000004	C	6.29061510	-2.36225263	1.17768005
C	7.69622654	-4.34101373	-0.00000005	C	5.95170732	-2.87286820	2.57220181
C	8.25915395	-5.75125429	0.00000002	C	6.35493712	-1.92565932	3.69645662
C	9.77718544	-5.79734435	0.00000005	C	5.61678807	-0.59037040	3.69859952
C	10.33700221	-7.20877436	0.00000001	C	4.10616087	-0.70594888	3.82180137
C	11.85514214	-7.26083473	0.00000002	C	3.39857532	0.63796860	3.79226560
C	12.39902886	-8.67799518	0.00000000	C	1.88901800	0.51282971	3.88861061
H	-0.45332011	1.07142974	0.00000020	H	-0.18848984	1.35561665	-0.05158192
H	-0.46820225	-0.45659631	0.88088805	H	-0.25978656	-0.08050926	0.96896389
H	-0.46820232	-0.45659605	-0.88088808	H	-0.39074455	-0.23744774	-0.78124803
H	1.82855933	0.56452742	-0.87479545	H	1.97576210	0.58617853	-1.02129247
H	1.82855942	0.56452745	0.87479534	H	2.10214030	0.73051362	0.71791203
H	1.64048684	-1.92586780	-0.87530265	H	1.60885770	-1.87194811	-0.79559876
H	1.64048682	-1.92586777	0.87530262	H	1.70939423	-1.72903898	0.94595843
H	3.91458609	-0.88642657	0.87544766	H	4.03038701	-0.85087215	0.74360769
H	3.91458609	-0.88642662	-0.87544769	H	3.96021334	-1.02906223	-0.99551500
H	3.71981634	-3.37871238	-0.87535208	H	3.60780518	-3.47626051	-0.75690448
H	3.71981637	-3.37871236	0.87535213	H	3.61645189	-3.32702551	0.97990450
H	5.99561455	-2.34416617	0.87540105	H	5.94555182	-2.73704289	-0.90612011
H	5.99561452	-2.34416616	-0.87540110	H	5.77912561	-4.15601298	0.10621554
H	5.79954685	-4.83640703	-0.87538067	H	6.07448867	-1.29319826	1.11033936
H	5.79954684	-4.83640704	0.87538057	H	7.36967539	-2.45334771	1.01751092
H	8.07453125	-3.79996466	0.87540486	H	6.43897880	-3.84122586	2.72478660
H	8.07453123	-3.79996473	-0.87540501	H	4.87945322	-3.07034033	2.64557290
H	7.88085521	-6.29239852	-0.87536040	H	7.43224733	-1.73540436	3.64102668
H	7.88085518	-6.29239842	0.87536049	H	6.18298400	-2.42396072	4.65653440
H	10.15623165	-5.25683817	0.87545531	H	5.85724544	-0.02855230	2.78967810
H	10.15623166	-5.25683811	-0.87545517	H	5.99476135	0.01810283	4.52709810
H	9.95747946	-7.74937554	-0.87531716	H	3.85431108	-1.23195627	4.75086681
H	9.95747943	-7.74937562	0.87531712	H	3.70905668	-1.32340863	3.00962987
H	12.23347091	-6.72101359	0.87480185	H	3.66574573	1.16041140	2.86662780
H	12.23347093	-6.72101357	-0.87480178	H	3.77184159	1.26449221	4.60948113
H	13.49041385	-8.69135032	-0.00000002	H	1.39959379	1.48801988	3.85554780
H	12.05990998	-9.22856445	-0.88090053	H	1.59403578	0.02416994	4.82044058
H	12.05991001	-9.22856448	0.88090053	H	1.49222041	-0.08518690	3.06436147

$C_{14}H_{30}$ Linear Conformer				$C_{14}H_{30}$ Hairpin Conformer			
44				44			
-14997.506952522 eV				-14997.514181841 eV			
C	-0.07354907	0.05469594	-0.00000010	C	0.20009471	0.45775748	0.16626120
C	1.44405696	0.02893430	0.00000006	C	1.69409165	0.25086846	-0.00099593
C	2.01255965	-1.37959589	0.00000009	C	2.10653631	-1.20879844	0.08015073
C	3.53024346	-1.42233887	0.00000001	C	3.60685239	-1.41696255	-0.02321482
C	4.09283739	-2.83281601	0.00000001	C	4.01749554	-2.87600878	0.09187186
C	5.61049063	-2.88011167	-0.00000002	C	5.52445550	-3.11336317	0.06589441
C	6.17034199	-4.29167706	-0.00000002	C	6.29676666	-2.41368123	1.17870404
C	7.68806646	-4.34031755	-0.00000000	C	5.95400488	-2.90712691	2.57911507
C	8.24785582	-5.75195139	0.00000000	C	6.37324313	-1.95631011	3.69467669
C	9.76558262	-5.79950244	0.00000004	C	5.65781121	-0.60870286	3.68366309
C	10.32808635	-7.21015977	0.00000004	C	4.14574952	-0.69625665	3.81263312
C	11.84589783	-7.25303247	-0.00000000	C	3.45930918	0.65492548	3.72216057
C	12.41452532	-8.66158520	0.00000000	C	1.94575188	0.56884849	3.81719208
C	13.93222169	-8.68759474	-0.00000004	C	1.26164491	1.91452823	3.66270320
H	-0.46003859	1.07541708	-0.00000013	H	-0.07144675	1.51266731	0.09616516
H	-0.47457927	-0.45260459	0.88088315	H	-0.14009238	0.09307569	1.13877852
H	-0.47457904	-0.45260456	-0.88088346	H	-0.36040153	-0.08184527	-0.60113624
H	1.82192455	0.56911291	-0.87478508	H	2.01991077	0.66430302	-0.96156972
H	1.82192439	0.56911290	0.87478527	H	2.23079666	0.81647091	0.76839740
H	1.63455802	-1.92134228	-0.87528428	H	1.59809804	-1.77627542	-0.70823471
H	1.63455810	-1.92134222	0.87528452	H	1.75182078	-1.63079795	1.02895389
H	3.90833678	-0.88114083	0.87544734	H	4.09078661	-0.82903871	0.76311775
H	3.90833669	-0.88114077	-0.87544731	H	3.97174214	-1.00806781	-0.97325879
H	3.71369924	-3.37344245	-0.87532076	H	3.55828761	-3.44037533	-0.72667920
H	3.71369927	-3.37344241	0.87532081	H	3.59006518	-3.29331907	1.00998640
H	5.98993835	-2.33983313	0.87540484	H	5.91739000	-2.77984821	-0.90047907
H	5.98993831	-2.33983315	-0.87540490	H	5.71457251	-4.19113162	0.11536706
H	5.79064394	-4.83186595	-0.87536393	H	6.11819594	-1.33809816	1.10886351
H	5.79064397	-4.83186594	0.87536390	H	7.37045700	-2.54293700	1.00850887
H	8.06773468	-3.80011812	0.87536708	H	6.42743491	-3.88116791	2.73905271
H	8.06773474	-3.80011812	-0.87536707	H	4.87957848	-3.08928934	2.65685949
H	7.86833676	-6.29216930	-0.87540875	H	7.45365463	-1.78447169	3.63736999
H	7.86833671	-6.29216932	0.87540873	H	6.19396739	-2.44289319	4.65938067
H	10.14481866	-5.25894795	0.87532920	H	5.90458032	-0.06310585	2.76645842
H	10.14481868	-5.25894796	-0.87532912	H	6.04964852	0.00427464	4.50230753
H	9.94994757	-7.75130762	-0.87545641	H	3.88702009	-1.17885660	4.76293625
H	9.94994761	-7.75130760	0.87545651	H	3.74001122	-1.34161290	3.02711893
H	12.22391905	-6.71133096	0.87529832	H	3.73378779	1.13221421	2.77296840
H	12.22391901	-6.71133103	-0.87529838	H	3.83767102	1.31676489	4.51012778
H	12.03682305	-9.20186605	-0.87479190	H	1.67015112	0.12155708	4.77832085
H	12.03682310	-9.20186605	0.87479193	H	1.57870962	-0.11930799	3.04800438
H	14.31825253	-9.70852204	-0.00000006	H	0.17657838	1.82704389	3.74311095
H	14.33355455	-8.18056884	0.88089598	H	1.48585875	2.35938468	2.68990983
H	14.33355450	-8.18056884	-0.88089609	H	1.59657969	2.61691024	4.42994367

$C_{15}H_{32}$ Linear Conformer				$C_{15}H_{32}$ Hairpin Conformer			
47				47			
-16066.440468400 eV				-16066.461140030 eV			
C	-0.08011504	0.05914204	0.00000005	C	-0.26221804	-0.30011338	0.02008842
C	1.43747278	0.03338873	-0.00000000	C	1.24611650	-0.22469380	-0.13036446
C	2.00594824	-1.37512908	-0.00000005	C	1.92912154	-1.57050360	0.04082011
C	3.52361168	-1.41798164	0.00000001	C	3.44141495	-1.50162919	-0.07566699
C	4.08596568	-2.82855342	-0.00000001	C	4.11623246	-2.85691162	0.03993290
C	5.60355600	-2.87593315	-0.00000001	C	5.62747845	-2.78692900	-0.10891944
C	6.16398820	-4.28730460	-0.00000000	C	6.32953809	-4.14097220	-0.07254526
C	7.68174317	-4.33400815	-0.00000001	C	5.90955004	-5.10283876	-1.17833952
C	8.24456925	-5.74435062	-0.00000003	C	6.28774135	-4.64003621	-2.57969975
C	9.76258148	-5.78835726	-0.00000002	C	5.53603622	-5.35486910	-3.69688805
C	10.32638974	-7.19822884	-0.00000002	C	4.02780546	-5.12401837	-3.69791586
C	11.84442110	-7.24411575	0.00000002	C	3.61438309	-3.66522320	-3.80497751
C	12.40390632	-8.65570265	0.00000004	C	2.10984571	-3.46573703	-3.76449659
C	13.92199858	-8.70837831	-0.00000003	C	1.69422886	-2.00585063	-3.82000261
C	14.46548417	-10.12566399	0.00000004	C	0.19122435	-1.81334235	-3.74063789
H	-0.46660034	1.07986921	0.00000021	H	-0.72932754	0.67775165	-0.11017933
H	-0.48114419	-0.44816240	0.88088462	H	-0.53967557	-0.67141748	1.00957487
H	-0.48114429	-0.44816215	-0.88088462	H	-0.69769768	-0.97616008	-0.71925823
H	1.81534443	0.57356715	-0.87478938	H	1.49795081	0.18292370	-1.11572089
H	1.81534448	0.57356712	0.87478938	H	1.65418118	0.48099342	0.60154270
H	1.62795689	-1.91688478	-0.87528686	H	1.53993943	-2.27019557	-0.70887140
H	1.62795685	-1.91688485	0.87528671	H	1.65852684	-1.99290949	1.01584310
H	3.90177646	-0.87681845	0.87544630	H	3.83642911	-0.82586167	0.69201780
H	3.90177654	-0.87681842	-0.87544624	H	3.70948152	-1.05024086	-1.03903986
H	3.70673430	-3.36908969	-0.87533501	H	3.69447803	-3.51834915	-0.72343376
H	3.70673429	-3.36908966	0.87533501	H	3.86520681	-3.31246879	1.00536497
H	5.98302002	-2.33562796	0.87539103	H	6.03402416	-2.15758711	0.68967952
H	5.98302002	-2.33562801	-0.87539108	H	5.86863216	-2.26673668	-1.04211034
H	5.78464536	-4.82772324	-0.87538306	H	6.13734477	-4.61114359	0.89790894
H	5.78464535	-4.82772324	0.87538304	H	7.41207545	-3.98145440	-0.12434385
H	8.06048347	-3.79316291	0.87536696	H	4.83015976	-5.26100718	-1.11720905
H	8.06048342	-3.79316291	-0.87536701	H	6.35973286	-6.08332338	-0.99348585
H	7.86638749	-6.28558170	-0.87538611	H	7.36384643	-4.78082425	-2.72297210
H	7.86638747	-6.28558173	0.87538604	H	6.11890836	-3.56464039	-2.67428033
H	10.14045814	-5.24696029	0.87539566	H	5.72975514	-6.43121271	-3.63421333
H	10.14045814	-5.24696026	-0.87539569	H	5.94316211	-5.02800739	-4.65983217
H	9.94827539	-7.73954304	-0.87534501	H	3.58385990	-5.55270844	-2.79314394
H	9.94827535	-7.73954305	0.87534496	H	3.58759529	-5.68127619	-4.53152712
H	12.22355223	-6.70364650	0.87545606	H	4.01815780	-3.23747383	-4.73069690
H	12.22355224	-6.70364650	-0.87545602	H	4.06063138	-3.08979931	-2.98763496
H	12.02414684	-9.19618000	-0.87530310	H	1.71320792	-3.91654760	-2.84615846
H	12.02414683	-9.19617998	0.87530321	H	1.64165009	-4.01095763	-4.59259774
H	14.30057835	-8.16871023	0.87479657	H	2.07635878	-1.55619459	-4.74273420
H	14.30057836	-8.16871032	-0.87479671	H	2.17747825	-1.46647244	-2.99838021
H	15.55688513	-10.13917501	0.00000004	H	-0.08258684	-0.75682801	-3.75680937
H	14.12635483	-10.67623480	-0.88090027	H	-0.21032930	-2.24581578	-2.82083220
H	14.12635483	-10.67623470	0.88090040	H	-0.31310543	-2.30126034	-4.57831981

**$C_{16}H_{34}$ Linear Conformer**				**$C_{16}H_{34}$ Hairpin Conformer**			
50				50			
-17135.374217936 eV				-17135.422062787 eV			
C	-0.08679988	0.06340140	-0.00000010	C	-0.27562490	-0.34050631	0.01375086
C	1.43075424	0.03773848	0.00000003	C	1.23536516	-0.24955310	-0.09420693
C	1.99944264	-1.37067555	0.00000012	C	1.92514580	-1.59659088	0.03343309
C	3.51709688	-1.41313802	-0.00000001	C	3.43753820	-1.51134401	-0.06786527
C	4.07982845	-2.82353093	-0.00000002	C	4.12653911	-2.86091049	0.02714878
C	5.59742238	-2.87055828	-0.00000003	C	5.63821373	-2.76894303	-0.10327632
C	6.15780815	-4.28189013	0.00000002	C	6.35928305	-4.11296987	-0.07332004
C	7.67556219	-4.32945566	0.00000007	C	5.94554497	-5.07975923	-1.17678919
C	8.23630536	-5.74059524	0.00000003	C	6.28563974	-4.60058313	-2.58236524
C	9.75412569	-5.78817280	0.00000001	C	5.53982848	-5.33809281	-3.68834917
C	10.31457099	-7.19958817	-0.00000002	C	4.02679234	-5.14418456	-3.67291142
C	11.83230676	-7.24689818	0.00000001	C	3.57350608	-3.70012935	-3.81385690
C	12.39503886	-8.65752336	-0.00000005	C	2.06589446	-3.54001127	-3.73333763
C	13.91289474	-8.70010002	-0.00000000	C	1.60307951	-2.09878640	-3.84937385
C	14.48185992	-10.10851625	0.00000000	C	0.09692945	-1.93908293	-3.73675464
C	15.99954868	-10.13446743	-0.00000003	C	-0.36157669	-0.49785066	-3.86148044
H	-0.47329522	1.08413171	-0.00000024	H	-0.74606444	0.63979830	-0.08247087
H	-0.48784162	-0.44389425	0.88088623	H	-0.57551231	-0.75977426	0.97733377
H	-0.48784144	-0.44389442	-0.88088641	H	-0.68828422	-0.98321013	-0.76736067
H	1.80858864	0.57796308	-0.87478342	H	1.51070351	0.20359300	-1.05310554
H	1.80858852	0.57796318	0.87478348	H	1.61973551	0.42576067	0.67775003
H	1.62156320	-1.91252471	-0.87528458	H	1.54739422	-2.27089064	-0.74463065
H	1.62156335	-1.91252458	0.87528497	H	1.64929429	-2.05585738	0.99011515
H	3.89512443	-0.87186430	0.87544306	H	3.81874832	-0.84705373	0.71661672
H	3.89512428	-0.87186433	-0.87544317	H	3.70883587	-1.03777138	-1.01969906
H	3.70071719	-3.36418321	-0.87532526	H	3.72171773	-3.51174058	-0.75425694
H	3.70071721	-3.36418320	0.87532523	H	3.87067323	-3.33895950	0.98036702
H	5.97679192	-2.33019803	0.87540112	H	6.02694279	-2.14292107	0.70667681
H	5.97679193	-2.33019804	-0.87540116	H	5.88278052	-2.23530020	-1.02781252
H	5.77835655	-4.82229363	-0.87535853	H	6.18071611	-4.58737400	0.89747365
H	5.77835647	-4.82229352	0.87535860	H	7.43900425	-3.93761373	-0.13174520
H	8.05474536	-3.78892400	0.87537944	H	4.87213451	-5.26716255	-1.09694006
H	8.05474543	-3.78892395	-0.87537924	H	6.42507359	-6.04894637	-1.00564171
H	7.85708485	-6.28111120	-0.87537886	H	7.36356650	-4.70518797	-2.74262041
H	7.85708487	-6.28111126	0.87537889	H	6.07990698	-3.53125236	-2.67101002
H	10.13350718	-5.24772782	0.87536165	H	5.76048148	-6.40896582	-3.62202616
H	10.13350714	-5.24772774	-0.87536161	H	5.92795690	-5.00744388	-4.65757503
H	9.93515572	-7.73989466	-0.87540704	H	3.60734812	-5.55924065	-2.75031856
H	9.93515571	-7.73989466	0.87540701	H	3.58823741	-5.73445578	-4.48428554
H	12.21153559	-6.70633002	0.87533471	H	3.93700346	-3.29446543	-4.76566888
H	12.21153565	-6.70632993	-0.87533460	H	4.02930884	-3.08578369	-3.03096918
H	12.01698774	-9.19875969	-0.87545392	H	1.71275814	-3.95633152	-2.78159175
H	12.01698768	-9.19875977	0.87545376	H	1.58962471	-4.13997512	-4.51769645
H	14.29077809	-8.15829677	0.87530229	H	1.94117565	-1.68330339	-4.80610230
H	14.29077814	-8.15829678	-0.87530228	H	2.08924752	-1.49806502	-3.07120386
H	14.10429700	-10.64891517	-0.87479055	H	-0.23530727	-2.35081882	-2.77722652
H	14.10429702	-10.64891517	0.87479057	H	-0.38764927	-2.54766501	-4.50778539
H	16.38544162	-11.15547267	-0.00000003	H	-1.44636278	-0.41150935	-3.77555795
H	16.40101189	-9.62755217	0.88090333	H	-0.07012189	-0.07559969	-4.82634194
H	16.40101184	-9.62755219	-0.88090343	H	0.08235453	0.12565104	-3.08194570

$C_{17}H_{36}$ Linear Conformer				$C_{17}H_{36}$ Hairpin Conformer			
53				53			
-18204.307731788 eV				-18204.372288763 eV			
C	-0.09335135	0.06780709	0.00000006	C	0.17536522	0.41256199	0.08940347
C	1.42417142	0.04220069	-0.00000002	C	1.68255385	0.27221896	-0.02049656
C	1.99298993	-1.36613572	-0.00000001	C	2.15293980	-1.16855690	0.07797148
C	3.51063606	-1.40845347	0.00000004	C	3.65887087	-1.32538509	-0.03156196
C	4.07327391	-2.81885537	-0.00000000	C	4.12234309	-2.76817829	0.06231091
C	5.59082198	-2.86622236	-0.00000000	C	5.63052880	-2.92441149	-0.01730118
C	6.15074778	-4.27773813	0.00000001	C	6.08718539	-4.36883456	0.10829578
C	7.66842362	-4.32545690	-0.00000007	C	7.60047036	-4.55973986	0.07813047
C	8.22988067	-5.73633448	-0.00000004	C	8.35595119	-3.82146258	1.17719782
C	9.74773287	-5.78160144	-0.00000003	C	8.03403716	-4.30566579	2.58496020
C	10.31164162	-7.19152386	-0.00000003	C	8.46132048	-3.34356749	3.68766609
C	11.82969036	-7.23494453	-0.00000001	C	7.74315884	-1.99773027	3.67015110
C	12.39364282	-8.64479320	0.00000002	C	6.23255153	-2.08769862	3.81367995
C	13.91169602	-8.69104364	0.00000006	C	5.54386298	-0.73775183	3.72184752
C	14.47067295	-10.10288594	0.00000001	C	4.03268833	-0.82230636	3.84028210
C	15.98874416	-10.15615261	0.00000000	C	3.33998508	0.52162612	3.69732525
C	16.53209512	-11.57347069	0.00000003	C	1.83029714	0.42838951	3.82027635
H	-0.47983463	1.08854962	0.00000017	H	-0.13889839	1.45514740	0.01465475
H	-0.49441863	-0.43947245	0.88088699	H	-0.18698625	0.02547004	1.04485888
H	-0.49441872	-0.43947229	-0.88088692	H	-0.32978957	-0.14479053	-0.70321017
H	1.80197828	0.58245820	-0.87478147	H	2.02382951	0.69649263	-0.97078295
H	1.80197838	0.58245825	0.87478137	H	2.16465609	0.86298532	0.76532139
H	1.61518345	-1.90805540	-0.87528011	H	1.66800295	-1.76100030	-0.70715589
H	1.61518343	-1.90805538	0.87528010	H	1.81272794	-1.59669557	1.02882882
H	3.88863892	-0.86714988	0.87544316	H	4.14256171	-0.73856631	0.75855475
H	3.88863900	-0.86714984	-0.87544302	H	4.00091919	-0.89458211	-0.97993208
H	3.69408949	-3.35946808	-0.87532234	H	3.64886939	-3.35596703	-0.73292631
H	3.69408946	-3.35946817	0.87532226	H	3.76744125	-3.19990885	1.00640292
H	5.97038135	-2.32596452	0.87539448	H	6.08328054	-2.31790390	0.77358511
H	5.97038132	-2.32596455	-0.87539451	H	5.99485093	-2.50668561	-0.96371910
H	5.77113602	-4.81801949	-0.87536820	H	5.64321003	-4.95329362	-0.70448194
H	5.77113607	-4.81801945	0.87536827	H	5.67574446	-4.79194068	1.03087573
H	8.04762515	-3.78487611	0.87535467	H	7.97806437	-4.22697586	-0.89461053
H	8.04762503	-3.78487618	-0.87535491	H	7.82383593	-5.63020731	0.14134562
H	7.85110081	-6.27715765	-0.87539238	H	8.14376302	-2.75285432	1.09344045
H	7.85110078	-6.27715763	0.87539229	H	9.43215665	-3.92084327	1.00355470
H	10.12602792	-5.24038249	0.87535380	H	8.51596331	-5.27510222	2.74679785
H	10.12602794	-5.24038249	-0.87535385	H	6.96157967	-4.49346544	2.67724432
H	9.93372293	-7.73298377	-0.87537204	H	9.54080082	-3.17052234	3.61934099
H	9.93372291	-7.73298379	0.87537195	H	8.29044113	-3.82149317	4.65839725
H	12.20745404	-6.69345071	0.87539931	H	7.98051141	-1.46031068	2.74589817
H	12.20745402	-6.69345073	-0.87539936	H	8.14041230	-1.37631177	4.47964843
H	12.01547886	-9.18611724	-0.87533160	H	5.98401786	-2.56464519	4.76946084
H	12.01547881	-9.18611724	0.87533162	H	5.82027352	-2.73881090	3.03627293
H	14.29103405	-8.15069555	0.87545494	H	5.80437376	-0.26861167	2.76491412
H	14.29103406	-8.15069546	-0.87545477	H	5.93436377	-0.07052125	4.49923348
H	14.09067248	-10.64322003	-0.87529876	H	3.76704961	-1.26653434	4.80680506
H	14.09067249	-10.64322009	0.87529876	H	3.64775952	-1.50920508	3.07685984
H	16.36754688	-9.61660997	0.87478891	H	3.60537132	0.95880592	2.72842032
H	16.36754688	-9.61661000	-0.87478893	H	3.73002941	1.21037644	4.45450133
H	17.62352630	-11.58682702	0.00000007	H	1.35372234	1.40257193	3.69616356
H	16.19314530	-12.12414593	-0.88090692	H	1.53997993	0.03825148	4.79871264
H	16.19314525	-12.12414592	0.88090697	H	1.41595443	-0.24147032	3.06339590

$C_{18}H_{38}$ Linear Conformer				$C_{18}H_{38}$ Hairpin Conformer			
56				56			
-19273.241483473 eV				-19273.336106752 eV			
C	-0.09996435	0.07243623	-0.00000010	C	0.25743607	0.53988575	0.17788536
C	1.41772887	0.04646779	0.00000003	C	1.75501970	0.35366389	0.01838741
C	1.98628454	-1.36202456	0.00000008	C	2.18954410	-1.09822502	0.11722135
C	3.50401346	-1.40436286	0.00000002	C	3.68894449	-1.29125036	-0.02085081
C	4.06700807	-2.81461304	-0.00000001	C	4.12328391	-2.74204628	0.08653548
C	5.58459326	-2.86136132	-0.00000002	C	5.62633853	-2.93007429	-0.01546929
C	6.14502397	-4.27263557	0.00000002	C	6.05739410	-4.38110382	0.12321261
C	7.66267607	-4.32000030	-0.00000002	C	7.56697316	-4.59924774	0.08297037
C	8.22388030	-5.73087713	0.00000001	C	8.34374991	-3.86790388	1.17167993
C	9.74167144	-5.77733666	0.00000006	C	8.02065214	-4.33387566	2.58562459
C	10.30298287	-7.18820771	0.00000000	C	8.47022413	-3.36903267	3.67675730
C	11.82072545	-7.23557078	-0.00000003	C	7.77235478	-2.01241775	3.65278228
C	12.38122142	-8.64693900	0.00000000	C	6.26174784	-2.07807551	3.80848265
C	13.89892135	-8.69393249	-0.00000001	C	5.59339912	-0.71791574	3.71930115
C	14.46196081	-10.10433842	-0.00000005	C	4.08141407	-0.77995804	3.84167952
C	15.97983576	-10.14672068	0.00000000	C	3.40979775	0.57564424	3.71527193
C	16.54852159	-11.55524684	0.00000002	C	1.89643485	0.51356190	3.82696167
C	18.06629835	-11.58150290	-0.00000002	C	1.23129249	1.86954901	3.67971054
H	-0.48626399	1.09319838	-0.00000010	H	-0.02877715	1.59023477	0.09842269
H	-0.50100864	-0.43479961	0.88087881	H	-0.08269737	0.17771326	1.15124679
H	-0.50100851	-0.43479956	-0.88087911	H	-0.29125656	-0.01320106	-0.58854071
H	1.79568080	0.58652386	-0.87478362	H	2.07658087	0.75940897	-0.94695728
H	1.79568065	0.58652387	0.87478373	H	2.28137238	0.93707955	0.78112759
H	1.60851372	-1.90388279	-0.87528822	H	1.67409971	-1.68413433	-0.65315085
H	1.60851377	-1.90388273	0.87528845	H	1.85898588	-1.51081141	1.07830741
H	3.88194327	-0.86309948	0.87544379	H	4.20124499	-0.70330605	0.75014222
H	3.88194321	-0.86309946	-0.87544377	H	4.02112152	-0.88226691	-0.98233405
H	3.68813911	-3.35536808	-0.87532364	H	3.62405325	-3.33129661	-0.69155077
H	3.68813913	-3.35536811	0.87532361	H	3.77558936	-3.15220834	1.04291714
H	5.96391198	-2.32102467	0.87539487	H	6.10395389	-2.32163466	0.75910866
H	5.96391199	-2.32102469	-0.87539491	H	5.98307168	-2.53302956	-0.97354874
H	5.76560959	-4.81298082	-0.87536015	H	5.59616706	-4.96700391	-0.67883204
H	5.76560967	-4.81298070	0.87536031	H	5.64658084	-4.78614362	1.05424731
H	8.04175696	-3.77946761	0.87537387	H	7.94314593	-4.27934624	-0.89469540
H	8.04175693	-3.77946765	-0.87537394	H	7.77174528	-5.67320234	0.15101094
H	7.84494566	-6.27153657	-0.87537269	H	8.15372105	-2.79580475	1.08053461
H	7.84494561	-6.27153655	0.87537270	H	9.41694889	-3.99082912	0.99318163
H	10.12049243	-5.23660742	0.87537363	H	8.48663210	-5.31025798	2.75357043
H	10.12049248	-5.23660731	-0.87537342	H	6.94573791	-4.50253870	2.68533393
H	9.92386794	-7.72871398	-0.87537626	H	9.55168636	-3.21278115	3.59925352
H	9.92386800	-7.72871402	0.87537627	H	8.29962513	-3.83566650	4.65292022
H	12.20005775	-6.69517587	0.87536507	H	8.01084747	-1.48658088	2.72204753
H	12.20005771	-6.69517595	-0.87536519	H	8.18548077	-1.39037609	4.45382801
H	12.00183506	-9.18720000	-0.87540205	H	6.01349737	-2.54846390	4.76759705
H	12.00183510	-9.18719992	0.87540212	H	5.83328433	-2.72481300	3.03621534
H	14.27787413	-8.15325343	0.87533497	H	5.85815894	-0.25147165	2.76215624
H	14.27787415	-8.15325338	-0.87533496	H	5.99591198	-0.05730674	4.49612212
H	14.08405812	-10.64560212	-0.87545368	H	3.81228408	-1.23074856	4.80418167
H	14.08405806	-10.64560216	0.87545352	H	3.68448494	-1.45278102	3.07204735
H	16.35759107	-9.60489507	0.87530162	H	3.68398108	1.02640535	2.75360422
H	16.35759114	-9.60489508	-0.87530159	H	3.80295095	1.25044739	4.48497396
H	16.17070116	-12.09537950	-0.87479135	H	1.62576614	0.07277099	4.79256338
H	16.17070118	-12.09537948	0.87479141	H	1.50955702	-0.17039012	3.06424049
H	18.45213780	-12.60246699	-0.00000001	H	0.14573937	1.79735538	3.76802112
H	18.46762280	-11.07452855	0.88089197	H	1.45453478	2.31386929	2.70657899
H	18.46762276	-11.07452857	-0.88089203	H	1.58176163	2.56482522	4.44657526

$C_{19}H_{40}$ Linear Conformer				$C_{19}H_{40}$ Hairpin Conformer			
59				59			
-20342.175004601 eV				-20342.282333612 eV			
C	-0.10652764	0.07684592	0.00000008	C	-0.31949768	-0.32893133	0.01895622
C	1.41114719	0.05092326	-0.00000002	C	1.18434698	-0.28524194	-0.18155097
C	1.97984046	-1.35749920	-0.00000001	C	1.84965173	-1.63777998	0.00592047
C	3.49757133	-1.39963345	-0.00000001	C	3.36102608	-1.59351783	-0.13074443
C	4.06063124	-2.80983661	0.00000003	C	4.02330400	-2.95033707	0.02834387
C	5.57819717	-2.85669657	-0.00000001	C	5.53542089	-2.90162362	-0.09954027
C	6.13827478	-4.26809609	0.00000001	C	6.19478683	-4.26450544	0.01482925
C	7.65587464	-4.31595003	-0.00000004	C	7.70680276	-4.20985167	-0.13248989
C	8.21667848	-5.72699152	-0.00000002	C	8.39693506	-5.56965170	-0.08314904
C	9.73440217	-5.77328923	-0.00000001	C	7.96539553	-6.54040964	-1.17636977
C	10.29691105	-7.18369551	-0.00000004	C	8.34091586	-6.09500718	-2.58358072
C	11.81473491	-7.22821799	0.00000001	C	7.60314711	-6.83836383	-3.69138115
C	12.37885486	-8.63799947	-0.00000002	C	6.09052361	-6.63869505	-3.69341548
C	13.89685274	-8.68155855	-0.00000002	C	5.64591048	-5.19040729	-3.81643903
C	14.46026949	-10.09159025	-0.00000002	C	4.13910876	-5.02093084	-3.74176886
C	15.97821361	-10.13840951	0.00000006	C	3.68964828	-3.57386650	-3.83632985
C	16.53692617	-11.55037259	-0.00000002	C	2.18596107	-3.40178582	-3.71977366
C	18.05501171	-11.60334957	-0.00000002	C	1.73218906	-1.95530036	-3.80815975
C	18.59867158	-13.02065676	0.00000006	C	0.22766321	-1.79793562	-3.68978957
H	-0.49280875	1.09762081	0.00000023	H	-0.77389218	0.65256867	-0.12750226
H	-0.50759207	-0.43037269	0.88088084	H	-0.57074608	-0.66403541	1.02815003
H	-0.50759219	-0.43037245	-0.88088075	H	-0.79077146	-1.01968200	-0.68389443
H	1.78907208	0.59100546	-0.87478276	H	1.41134112	0.09143482	-1.18483331
H	1.78907221	0.59100553	0.87478263	H	1.62733609	0.43229797	0.51763997
H	1.60215081	-1.89942376	-0.87528685	H	1.43920698	-2.34676265	-0.72288027
H	1.60215081	-1.89942375	0.87528683	H	1.58759361	-2.03562745	0.99358612
H	3.87544042	-0.85832472	0.87544556	H	3.77111534	-0.89987381	0.61290884
H	3.87544041	-0.85832478	-0.87544562	H	3.62657698	-1.17498227	-1.10907889
H	3.68176469	-3.35060377	-0.87532033	H	3.61898230	-3.64032106	-0.72203241
H	3.68176472	-3.35060372	0.87532043	H	3.75453980	-3.37363252	1.00320698
H	5.95761243	-2.31642311	0.87539752	H	5.94451803	-2.22901610	0.66351373
H	5.95761236	-2.31642320	-0.87539764	H	5.80135128	-2.45589422	-1.06608070
H	5.75871141	-4.80834028	-0.87536052	H	5.76655690	-4.92072524	-0.74968276
H	5.75871147	-4.80834023	0.87536059	H	5.93831245	-4.71823070	0.97978622
H	8.03516580	-3.77553431	0.87536456	H	8.11767044	-3.57795628	0.66193171
H	8.03516571	-3.77553437	-0.87536472	H	7.95440284	-3.69946062	-1.06936986
H	7.83764649	-6.26756052	-0.87538740	H	8.20385098	-6.02684848	0.89319960
H	7.83764648	-6.26756051	0.87538736	H	9.48045286	-5.41975309	-0.14025114
H	10.11308333	-5.23240859	0.87534956	H	6.88513235	-6.69013119	-1.10900000
H	10.11308335	-5.23240854	-0.87534954	H	8.40981511	-7.52204404	-0.98312810
H	9.91845733	-7.72466028	-0.87538954	H	9.41937564	-6.22289427	-2.72151579
H	9.91845727	-7.72466034	0.87538940	H	8.15696723	-5.02367471	-2.69537496
H	12.19277834	-6.68693010	0.87536569	H	7.81831433	-7.90964636	-3.61538705
H	12.19277843	-6.68693010	-0.87536563	H	8.00341276	-6.51536039	-4.65843882
H	12.00096812	-9.17940719	-0.87536580	H	5.65703782	-7.06661865	-2.78331334
H	12.00096810	-9.17940729	0.87536569	H	5.66115502	-7.21444539	-4.52022570
H	14.27465266	-8.14018363	0.87540499	H	6.01822453	-4.77204614	-4.75945358
H	14.27465265	-8.14018363	-0.87540504	H	6.10003618	-4.59077173	-3.02123391
H	14.08181679	-10.63262977	-0.87533112	H	3.77809848	-5.44879016	-2.79823207
H	14.08181670	-10.63262982	0.87533102	H	3.66188759	-5.60441926	-4.53784025
H	16.35766990	-9.59823274	0.87545699	H	4.03178287	-3.14784552	-4.78681024
H	16.35766999	-9.59823258	-0.87545675	H	4.18247812	-2.99061638	-3.04925497
H	16.15685381	-12.09058033	-0.87530102	H	1.84564562	-3.82919264	-2.76850655
H	16.15685383	-12.09058041	0.87530095	H	1.69102165	-3.98583130	-4.50499789
H	18.43346305	-11.06365290	0.87479080	H	2.07456315	-1.52777823	-4.75665870
H	18.43346307	-11.06365297	-0.87479086	H	2.22565266	-1.37710489	-3.02020018
H	19.69004218	-13.03400068	0.00000014	H	-0.07370887	-0.74950058	-3.72838129

H	18.25959499	-13.57120304	-0.88089654	H	-0.13621383	-2.21247330	-2.74656940
H	18.25959488	-13.57120300	0.88089665	H	-0.28750533	-2.32285869	-4.49797375

$C_{20}H_{42}$ Linear Conformer				$C_{20}H_{42}$ Hairpin Conformer			
62				62			
-21411.108749417 eV				-21411.242104690 eV			
C	-0.11324316	0.08121166	-0.00000011	C	-0.31160727	-0.35224873	0.00133180
C	1.40443080	0.05529430	0.00000006	C	1.19803805	-0.30662074	-0.14514245
C	1.97323820	-1.35307781	0.00000006	C	1.85310166	-1.67057429	-0.01395958
C	3.49098365	-1.39502660	0.00000001	C	3.36492361	-1.62507716	-0.14116528
C	4.05415987	-2.80518050	-0.00000003	C	4.03136395	-2.98093032	0.00851693
C	5.57171606	-2.85197871	-0.00000005	C	5.54431196	-2.91830563	-0.10229354
C	6.13184145	-4.26336169	0.00000006	C	6.21866483	-4.27369503	0.01066950
C	7.64942971	-4.31100645	-0.00000001	C	7.73113048	-4.19708624	-0.12131446
C	8.21054232	-5.72189231	0.00000002	C	8.44065355	-5.54699280	-0.07991045
C	9.72828674	-5.76784067	-0.00000003	C	8.01691198	-6.52043505	-1.17360531
C	10.29057459	-7.17825328	-0.00000002	C	8.34923555	-6.05311545	-2.58481986
C	11.80837510	-7.22413637	0.00000003	C	7.60366937	-6.80511720	-3.68117068
C	12.36972361	-8.63502409	0.00000008	C	6.08875393	-6.62653457	-3.65698590
C	13.88746145	-8.68271063	0.00000001	C	5.61898823	-5.18819483	-3.80289461
C	14.44768498	-10.09424611	-0.00000004	C	4.11092917	-5.04413125	-3.70435326
C	15.96541417	-10.14140838	-0.00000002	C	3.62806901	-3.60994591	-3.82794440
C	16.52869125	-11.55177160	-0.00000005	C	2.12224000	-3.47280520	-3.69223750
C	18.04664812	-11.59375043	-0.00000002	C	1.62378468	-2.04592682	-3.83216361
C	18.61571973	-13.00213739	0.00000000	C	0.11445798	-1.92304775	-3.71524730
C	20.13351362	-13.02837495	-0.00000002	C	-0.38343548	-0.49860220	-3.87545214
H	-0.49947266	1.10201062	-0.00000005	H	-0.75565961	0.64038343	-0.09383454
H	-0.51433059	-0.42597899	0.88088459	H	-0.59835510	-0.75218036	0.97694392
H	-0.51433041	-0.42597880	-0.88088500	H	-0.76295645	-0.99063482	-0.76160720
H	1.78233633	0.59538549	-0.87478569	H	1.46249853	0.12634851	-1.11611263
H	1.78233613	0.59538539	0.87478595	H	1.62033895	0.36708385	0.60820800
H	1.59563447	-1.89505558	-0.87529156	H	1.44326046	-2.34468430	-0.77519539
H	1.59563451	-1.89505557	0.87529170	H	1.58341547	-2.10920667	0.95418580
H	3.86878794	-0.85368043	0.87544997	H	3.76990535	-0.93860207	0.61182087
H	3.86878789	-0.85368039	-0.87544994	H	3.63484603	-1.19545182	-1.11368213
H	3.67533925	-3.34596684	-0.87532655	H	3.63938797	-3.66484596	-0.75367224
H	3.67533928	-3.34596689	0.87532648	H	3.75516204	-3.41663693	0.97587772
H	5.95114319	-2.31171993	0.87540163	H	5.93752741	-2.24638038	0.66971696
H	5.95114318	-2.31172004	-0.87540180	H	5.81658308	-2.46287307	-1.06269900
H	5.75231958	-4.80362682	-0.87536633	H	5.80726106	-4.93081172	-0.76205670
H	5.75231972	-4.80362664	0.87536661	H	5.95852886	-4.73653070	0.97024570
H	8.02863672	-3.77054661	0.87537122	H	8.12598567	-3.56744311	0.68281281
H	8.02863666	-3.77054668	-0.87537132	H	7.98008271	-3.67387057	-1.05061463
H	7.83166152	-6.26258453	-0.87538283	H	8.25900678	-6.01102074	0.89543786
H	7.83166160	-6.26258449	0.87538295	H	9.52166008	-5.38101422	-0.14122433
H	10.10685589	-5.22692027	0.87536938	H	6.94340147	-6.70340864	-1.08535889
H	10.10685582	-5.22692020	-0.87536942	H	8.49445674	-7.48985717	-0.99814948
H	9.91192548	-7.71911926	-0.87537064	H	9.42712050	-6.15336322	-2.74817096
H	9.91192544	-7.71911916	0.87537064	H	8.13694814	-4.98591465	-2.68309210
H	12.18710014	-6.68334370	0.87538381	H	7.83527816	-7.87333698	-3.60984093
H	12.18710021	-6.68334374	-0.87538375	H	7.98218837	-6.47621636	-4.65488352
H	11.99049441	-9.17546374	-0.87537258	H	5.67986567	-7.04077617	-2.72932506
H	11.99049450	-9.17546364	0.87537283	H	5.65122726	-7.22611097	-4.46203690
H	14.26691000	-8.14240311	0.87537152	H	5.96619514	-4.78564201	-4.76211028
H	14.26690994	-8.14240309	-0.87537151	H	6.07781459	-4.56326155	-3.03017835
H	14.06817296	-10.63441356	-0.87540892	H	3.77470225	-5.45590053	-2.74448559
H	14.06817295	-10.63441362	0.87540881	H	3.63160713	-5.65672876	-4.47708278

H 16.34435730 -9.60072759 0.87533849<br>H 16.34435735 -9.60072749 -0.87533844<br>H 16.15093970 -12.09313531 -0.87546133<br>H 16.15093967 -12.09313530 0.87546123<br>H 18.42421893 -11.05180229 0.87530711<br>H 18.42421899 -11.05180230 -0.87530712<br>H 18.23803300 -13.54236328 -0.87479429<br>H 18.23803301 -13.54236326 0.87479432<br>H 20.51916646 -14.04943113 -0.00000009<br>H 20.53497565 -12.52153115 0.88090144<br>H 20.53497561 -12.52153106 -0.88090144	H 3.94439980 -3.20165892 -4.79499390<br>H 4.11947767 -2.99446473 -3.06504675<br>H 1.80934749 -3.87304543 -2.72005170<br>H 1.63309905 -4.10132545 -4.44586089<br>H 1.94516407 -1.64239916 -4.79980771<br>H 2.09914824 -1.41716178 -3.07021970<br>H -0.20343991 -2.31889310 -2.74445869<br>H -0.35611343 -2.56391373 -4.46862611<br>H -1.47007585 -0.43983278 -3.78998400<br>H -0.10471501 -0.09382568 -4.85140364<br>H 0.04385892 0.15687356 -3.11317681

$C_{22}H_{46}$ Linear Conformer	$C_{22}H_{46}$ Hairpin Conformer
68<br>-23548.976111740 eV<br>C -0.15172933 0.11530899 -0.00000645<br>C 1.36579226 0.08447721 0.00000101<br>C 1.93040977 -1.32569666 0.00000648<br>C 3.44802600 -1.37118990 0.00000304<br>C 4.00979165 -2.78196457 0.00001252<br>C 5.52750630 -2.82861065 0.00002288<br>C 6.08888301 -4.23950292 0.00001186<br>C 7.60663344 -4.28588907 0.00001232<br>C 8.16868956 -5.69649840 -0.00004130<br>C 9.68647350 -5.74195516 -0.00007407<br>C 10.24954519 -7.15215938 -0.00029793<br>C 11.76736857 -7.19641873 -0.00030661<br>C 12.33162636 -8.60614408 -0.00082090<br>C 13.84948421 -8.64907068 -0.00066844<br>C 14.41507220 -10.05826395 -0.00153415<br>C 15.93298376 -10.09966513 -0.00081457<br>C 16.49992101 -11.50830413 -0.00196167<br>C 18.01790260 -11.54842057 0.00049334<br>C 18.58529640 -12.95690663 -0.00054641<br>C 20.10298581 -12.99708331 0.00360763<br>C 20.67258036 -14.40529046 0.00343906<br>C 22.19022639 -14.43036412 0.00702756<br>H -0.53460846 1.13738000 -0.00001211<br>H -0.55455789 -0.39050731 0.88088243<br>H -0.55454840 -0.39051333 -0.88089624<br>H 1.74538973 0.62348452 -0.87473852<br>H 1.74538192 0.62348830 0.87474153<br>H 1.55109311 -1.86648740 -0.87528170<br>H 1.55109819 -1.86647849 0.87530243<br>H 3.82679804 -0.83037780 0.87538413<br>H 3.82679432 -0.83039094 -0.87538776<br>H 3.63073176 -3.32262094 -0.87531232<br>H 3.63071961 -3.32261433 0.87533617<br>H 5.90652367 -2.28796015 0.87537739<br>H 5.90653497 -2.28794488 -0.87531755<br>H 5.70988508 -4.78017596 -0.87534137<br>H 5.70987966 -4.78019585 0.87535028<br>H 7.98547947 -3.74509711 0.87536002<br>H 7.98547514 -3.74504594 -0.87530623<br>H 7.78999794 -6.23735460 -0.87541453<br>H 7.79001687 -6.23743028 0.87529258<br>H 10.06500913 -5.20099280 0.87530565	68<br>-23549.154955869 eV<br>C -0.30524634 -0.43836852 -0.01911310<br>C 1.20765709 -0.37070778 -0.13321429<br>C 1.88311311 -1.72433819 -0.00901568<br>C 3.39514179 -1.65798990 -0.12347026<br>C 4.07247751 -3.00891799 0.01923899<br>C 5.58461955 -2.93815246 -0.09312830<br>C 6.26289363 -4.29174900 0.01686001<br>C 7.77457990 -4.21367835 -0.12279184<br>C 8.48391062 -5.56358268 -0.08320352<br>C 8.05177096 -6.53932579 -1.17187766<br>C 8.37871268 -6.07735064 -2.58603394<br>C 7.63301615 -6.83769758 -3.67681815<br>C 6.11823974 -6.65955010 -3.65244009<br>C 5.65022125 -5.22146935 -3.80621888<br>C 4.14244568 -5.07327735 -3.71126349<br>C 3.66706796 -3.63720391 -3.83745211<br>C 2.16185715 -3.48865277 -3.70936915<br>C 1.68260562 -2.05417195 -3.83736735<br>C 0.17568306 -1.90848718 -3.72809657<br>C -0.30271563 -0.47280837 -3.85624780<br>C -0.97620275 0.91493761 -0.16436852<br>C -1.80791071 -0.33090936 -3.72611556<br>H -0.57181221 -0.88048341 0.94699403<br>H -0.69113324 -1.12384973 -0.78079626<br>H 1.47939878 0.08338172 -1.09370681<br>H 1.59939674 0.30313716 0.63806811<br>H 1.49084894 -2.39699507 -0.78063806<br>H 1.61239355 -2.17716758 0.95213067<br>H 3.78670897 -0.97384278 0.63862210<br>H 3.66732759 -1.21725527 -1.09033458<br>H 3.68406885 -3.69179402 -0.74591111<br>H 3.79975263 -3.45127175 0.98462677<br>H 5.97639005 -2.26580267 0.67918234<br>H 5.85342335 -2.48071967 -1.05347511<br>H 5.84830962 -4.94963966 -0.75352475<br>H 6.00839427 -4.75482939 0.97790931<br>H 8.17272167 -3.58288796 0.67894323<br>H 8.01848719 -3.69136006 -1.05397311<br>H 8.30869594 -6.02530229 0.89440797<br>H 9.56460742 -5.39879051 -0.15250553<br>H 6.97810938 -6.71907309 -1.07771079<br>H 8.52775085 -7.50944198 -0.99612405

H	10.06495256	-5.20077220	-0.87534495	H	9.45658676	-6.17466875	-2.75124379
H	9.87127646	-7.69319426	-0.87574319	H	8.16219470	-5.01130837	-2.68898178
H	9.87128093	-7.69348644	0.87496938	H	7.86613421	-7.90512291	-3.59848228
H	12.14543843	-6.65532259	0.87519364	H	8.01024008	-6.51482117	-4.65306876
H	12.14542581	-6.65473075	-0.87544883	H	5.70956228	-7.06825341	-2.72219658
H	11.95392658	-9.14728149	-0.87644769	H	5.67965141	-7.26291490	-4.45418772
H	11.95374286	-9.14801371	0.87427234	H	6.00014949	-4.82431150	-4.76676214
H	14.22696984	-8.10791440	0.87504845	H	6.10841388	-4.59327235	-3.03575665
H	14.22718150	-8.10677237	-0.87558685	H	3.80294890	-5.48202359	-2.75135083
H	14.03822630	-10.59943073	-0.87750849	H	3.66262638	-5.68525495	-4.48409210
H	14.03742422	-10.60083622	0.87322082	H	3.99091370	-3.23030058	-4.80263821
H	16.30951718	-9.55854930	0.87533657	H	4.15816968	-3.02459858	-3.07183119
H	16.31053707	-9.55659514	-0.87530576	H	1.83928497	-3.89459429	-2.74276727
H	16.12458606	-12.04930194	-0.87868193	H	1.67059749	-4.10441758	-4.47203583
H	16.12180894	-12.05177330	0.87203628	H	2.01709754	-1.64515652	-4.79808072
H	18.39307999	-11.00750435	0.87732525	H	2.16263416	-1.44183376	-3.06512777
H	18.39616137	-11.00483149	-0.87335411	H	-0.15963432	-2.31865937	-2.76793918
H	18.21085432	-13.49766883	-0.87782042	H	-0.30559072	-2.52101995	-4.49983775
H	18.20619055	-13.50066112	0.87288676	H	0.02347677	-0.07183513	-4.82201680
H	20.47799998	-12.45617375	0.88068211	H	0.19158222	0.13834497	-3.09393325
H	20.48297883	-12.45386900	-0.86988346	H	-2.32501526	-0.90832913	-4.49629424
H	20.29285707	-14.94662206	0.87667738	H	-2.15471549	-0.69297939	-2.75526168
H	20.29710456	-14.94470249	-0.87282672	H	-2.12431707	0.70928578	-3.82222339
H	22.58900637	-13.92394417	0.88940808	H	-2.06153475	0.83792463	-0.07791278
H	22.57712405	-15.45091245	0.00673445	H	-0.62979625	1.60973062	0.60456465
H	22.59309346	-13.92193850	-0.87234731	H	-0.75345467	1.36217023	-1.13611671

**$C_{24}H_{50}$ Linear Conformer**				**$C_{24}H_{50}$ Hairpin Conformer**			
74				74			
-25686.843460850 eV				-25687.063069104 eV			
C	-0.20404761	0.15187096	-0.00003591	C	-0.23928155	-0.39584230	-0.01920257
C	1.31346324	0.12075312	-0.00001310	C	1.27381396	-0.36230959	-0.13495844
C	1.87784380	-1.28952699	0.00001559	C	1.92543532	-1.72861933	-0.01697773
C	3.39545715	-1.33527753	0.00004344	C	3.43818090	-1.68064155	-0.13689826
C	3.95707764	-2.74613225	0.00006522	C	4.10697669	-3.03535972	0.01107137
C	5.47480992	-2.79286896	0.00014240	C	5.61986540	-2.97096598	-0.10113117
C	6.03619229	-4.20376960	0.00015034	C	6.29485313	-4.32601205	0.01403656
C	7.55396558	-4.25021599	0.00027491	C	7.80725387	-4.24926064	-0.11826149
C	8.11579233	-5.66091349	0.00013610	C	8.51729285	-5.59903306	-0.07900900
C	9.63355791	-5.70693678	0.00022379	C	8.09282476	-6.57195876	-1.17299974
C	10.19579658	-7.11747366	-0.00032333	C	8.42358236	-6.10378550	-2.58402103
C	11.71355218	-7.16279988	-0.00033552	C	7.67751103	-6.85584188	-3.68007219
C	12.27689560	-8.57289954	-0.00156891	C	6.16263851	-6.67680001	-3.65573367
C	13.79470109	-8.61636595	-0.00152423	C	5.69316243	-5.23847899	-3.80251515
C	14.36051296	-10.02548992	-0.00365141	C	4.18517613	-5.09375129	-3.70229291
C	15.87842798	-10.06544546	-0.00299452	C	3.70363381	-3.65925890	-3.82852565
C	16.44833595	-11.47292727	-0.00561655	C	2.19786020	-3.52009115	-3.69551246
C	17.96642459	-11.50777106	-0.00245195	C	1.70111471	-2.09166446	-3.82946363
C	18.54118335	-12.91320213	-0.00404579	C	0.19094627	-1.97288207	-3.72647598
C	20.05943034	-12.94369368	0.00425038	C	-0.31943388	-0.54926836	-3.85630101
C	20.63669131	-14.34823338	0.00402094	C	-0.88180478	0.97678282	-0.12068161
C	22.15461343	-14.37667266	0.01963055	C	-1.83208934	-0.44132800	-3.76978477
C	22.73594933	-15.77999897	0.01718008	C	-2.33598472	0.98457707	-3.89582750
C	24.25370891	-15.79250770	0.03303286	C	-2.39467805	0.93637393	-0.00860611
H	-0.58671301	1.17402681	-0.00005609	H	-0.52095938	-0.85668924	0.93509461
H	-0.60700896	-0.35384198	0.88085597	H	-0.64971159	-1.04572909	-0.80078566
H	-0.60698306	-0.35386710	-0.88092522	H	1.55407415	0.09046164	-1.09377712
H	1.69314814	0.65970553	-0.87475337	H	1.68109609	0.29934735	0.63885171

H	1.69312226	0.65972992	0.87472343
H	1.49842507	-1.83024781	-0.87527503
H	1.49839630	-1.83022270	0.87530926
H	3.77429014	-0.79451048	0.87543062
H	3.77432419	-0.79452271	-0.87533652
H	3.57799748	-3.28674740	-0.87528077
H	3.57791138	-3.28675392	0.87536970
H	5.85380229	-2.25222768	0.87551657
H	5.85389306	-2.25220650	-0.87517958
H	5.65727930	-4.74444384	-0.87524411
H	5.65712767	-4.74450110	0.87544327
H	7.93278995	-3.70953342	0.87570404
H	7.93293044	-3.70933286	-0.87496987
H	7.73705372	-6.20161481	-0.87531738
H	7.73692207	-6.20186592	0.87537697
H	10.01229853	-5.16639306	0.87578276
H	10.01240269	-5.16571713	-0.87487152
H	9.81717679	-7.65804652	-0.87590777
H	9.81714718	-7.65877957	0.87479602
H	12.09203406	-6.62240304	0.87542920
H	12.09203068	-6.62094055	-0.87519747
H	11.89884827	-9.11336736	-0.87746433
H	11.89874051	-9.11500286	0.87326862
H	14.17232192	-8.07596348	0.87461134
H	14.17243676	-8.07332210	-0.87597533
H	13.98388533	-10.56600606	-0.88012391
H	13.98313512	-10.56898305	0.87065341
H	16.25422529	-9.52462329	0.87366558
H	16.25520022	-9.52083950	-0.87688715
H	16.07478009	-12.01387059	-0.88313378
H	16.07137055	-12.01834846	0.86766584
H	18.33875014	-10.96566877	0.87489165
H	18.34279167	-10.96178834	-0.87565772
H	18.17240236	-13.45500701	-0.88304445
H	18.16324115	-13.46038345	0.86770574
H	20.42742139	-12.40126916	0.88320747
H	20.43762496	-12.39651126	-0.86738291
H	20.25509956	-14.89680968	0.87333312
H	20.27281348	-14.88949704	-0.87736717
H	22.51834127	-13.83699700	0.90223231
H	22.53703216	-13.82601451	-0.84812798
H	22.35378801	-16.32889659	0.88462448
H	22.37220860	-16.31820442	-0.86475874
H	24.64124250	-15.28770392	0.92132222
H	24.64887294	-16.80990318	0.03041731
H	24.65954744	-15.27615107	-0.84033813
H	1.51981784	-2.39361622	-0.78827633
H	1.65188115	-2.17861848	0.94467093
H	3.83824121	-0.99592771	0.62044424
H	3.71120138	-1.24673753	-1.10664787
H	3.71583625	-3.71949353	-0.75149191
H	3.83251033	-3.47242382	0.97836674
H	6.01379198	-2.29701836	0.66883118
H	5.89083990	-2.51774962	-1.06295155
H	5.88344987	-4.98433903	-0.75777447
H	6.03453039	-4.78756882	0.97426593
H	8.20273689	-3.62064839	0.68659402
H	8.05567637	-3.72460989	-1.04700452
H	8.33754527	-6.06418270	0.89620771
H	9.59811557	-5.43221231	-0.14184078
H	7.01926297	-6.75491432	-1.08452230
H	8.57017063	-7.54149148	-0.99767201
H	9.50134897	-6.20239644	-2.74920490
H	8.20985640	-5.03665694	-2.68092235
H	7.90871945	-7.92418515	-3.60830300
H	8.05612840	-6.52749983	-4.65401469
H	5.75358827	-7.09022949	-2.72767419
H	5.72489295	-7.27711472	-4.46032258
H	6.03950325	-4.83670475	-4.76244615
H	6.15298535	-4.61289259	-3.03080322
H	3.85040452	-5.50287089	-2.74079685
H	3.70455696	-5.70836636	-4.47267027
H	4.02215234	-3.25268980	-4.79561685
H	4.19475926	-3.04363705	-3.06541537
H	1.88174387	-3.92422410	-2.72599053
H	1.70933240	-4.14415291	-4.45336414
H	2.03208449	-1.68115731	-4.79073408
H	2.16904226	-1.46944124	-3.05775396
H	-0.14015296	-2.39068813	-2.76799313
H	-0.27325003	-2.59545848	-4.50066611
H	0.01868085	-0.12720987	-4.81021602
H	0.13258297	0.07205892	-3.07443652
H	-2.28149460	-1.06142863	-4.55306997
H	-2.16831150	-0.86900888	-2.81887419
H	-0.47157401	1.62381812	0.66231551
H	-0.59426079	1.43789156	-1.07193767
H	-2.03501142	1.42367409	-4.85016134
H	-3.42477931	1.03418488	-3.83632222
H	-1.93264933	1.61665310	-3.10130867
H	-2.70555312	0.50400642	0.94562522
H	-2.83096857	1.93448989	-0.07859266
H	-2.83252093	0.32806705	-0.80340991

$C_{26}H_{54}$ Linear Conformer				$C_{26}H_{54}$ Hairpin Conformer			
80				80			
-27824.710842783 eV				-27824.973662093 eV			
C	-0.25288548	0.18611722	-0.00006882	C	-0.22278662	-0.46224674	-0.00467057
C	1.26462811	0.15489166	0.00000040	C	1.28926641	-0.40543554	-0.12854660
C	1.82887345	-1.25546212	0.00005046	C	1.95640827	-1.76434836	-0.01311031
C	3.34650061	-1.30155652	0.00014747	C	3.46841196	-1.70547105	-0.13369956
C	3.90775057	-2.71260797	0.00016682	C	4.14413181	-3.05694360	0.01377110
C	5.42550429	-2.75988856	0.00033561	C	5.65685885	-2.98654792	-0.09838813
C	5.98646414	-4.17100746	0.00025820	C	6.33556384	-4.33974947	0.01608455
C	7.50426156	-4.21793961	0.00048012	C	7.84757066	-4.26330215	-0.12250886
C	8.06572017	-5.62881049	0.00009724	C	8.55519377	-5.61430855	-0.08187320
C	9.58348729	-5.67526118	0.00029007	C	8.12491416	-6.58998274	-1.17150327
C	10.14532266	-7.08595764	-0.00073900	C	8.45211235	-6.12751938	-2.58544069
C	11.66304260	-7.13168998	-0.00061679	C	7.70574941	-6.88611843	-3.67714559
C	12.22606410	-8.54189467	-0.00261792	C	6.19056437	-6.70983961	-3.65164871
C	13.74382155	-8.58544381	-0.00224016	C	5.72101886	-5.27202042	-3.80455523
C	14.30979363	-9.99446484	-0.00539097	C	4.21344914	-5.12310511	-3.70494291
C	15.82768808	-10.03383454	-0.00394005	C	3.73764888	-3.68656794	-3.83091116
C	16.39851838	-11.44092781	-0.00800281	C	2.23247326	-3.53970136	-3.69807610
C	17.91660540	-11.47413707	-0.00369815	C	1.74619698	-2.10802133	-3.83221354
C	18.49406794	-12.87855813	-0.00768045	C	0.23741114	-1.97216376	-3.73156186
C	20.01236614	-12.90405587	0.00248133	C	-0.25079618	-0.54124514	-3.86935855
C	20.59683460	-14.30549522	0.00027081	C	-0.89224037	0.89560466	-0.11720805
C	22.11525440	-14.32492295	0.01869731	C	-1.75938894	-0.40272426	-3.77193827
C	22.70317235	-15.72508752	0.01796825	C	-2.24519911	1.02879210	-3.92043453
C	24.22110970	-15.74179926	0.04562711	C	-2.40412655	0.83652512	0.01692029
C	24.81388133	-17.14032381	0.04248199	C	-3.75169302	1.16376716	-3.79650732
C	26.33153607	-17.14045567	0.07124892	C	-3.06925258	2.19347112	-0.12245576
H	-0.63546742	1.20831073	-0.00010777	H	-0.48979700	-0.91916729	0.95566980
H	-0.65594926	-0.31954146	0.88081393	H	-0.62454607	-1.12796650	-0.77729943
H	-0.65587026	-0.31957892	-0.88096630	H	1.55866961	0.05058576	-1.08880032
H	1.64433845	0.69385115	-0.87473007	H	1.69120066	0.26164910	0.64326127
H	1.64425862	0.69388069	0.87474738	H	1.55604698	-2.43193546	-0.78498358
H	1.44936716	-1.79610450	-0.87525503	H	1.68716027	-2.21853120	0.94782241
H	1.44925994	-1.79608411	0.87532206	H	3.86492307	-1.01872461	0.62359036
H	3.72541947	-0.76088529	0.87556236	H	3.73873862	-1.27014683	-1.10355316
H	3.72553451	-0.76086605	-0.87520578	H	3.75583394	-3.74272204	-0.74878562
H	3.52852629	-3.25306847	-0.87521720	H	3.87145387	-3.49557148	0.98087771
H	3.52833044	-3.25312600	0.87543002	H	6.04792212	-2.31170262	0.67214945
H	5.80463483	-2.21944523	0.87577630	H	5.92600707	-2.53168800	-1.05987274
H	5.80483215	-2.21928635	-0.87492186	H	5.92177467	-5.00013725	-0.75257601
H	5.60745265	-4.71149481	-0.87521447	H	6.08044329	-4.79992264	0.97835663
H	5.60717787	-4.71171765	0.87547339	H	8.24651403	-3.63307222	0.67934044
H	7.88321149	-3.67754194	0.87603581	H	8.09333732	-3.74167860	-1.05360943
H	7.88346696	-3.67700979	-0.87463701	H	8.37718327	-6.07626258	0.89518413
H	7.68693764	-6.16925307	-0.87550316	H	9.63628440	-5.45068981	-0.14832263
H	7.68666463	-6.16988193	0.87518978	H	7.05143076	-6.77119856	-1.07819984
H	9.96236110	-5.13514691	0.87606335	H	8.60193465	-7.55951847	-0.99518456
H	9.96258953	-5.13385215	-0.87458492	H	9.52981317	-6.22597469	-2.75129658
H	9.76667243	-7.62610407	-0.87658130	H	8.23697275	-5.06111577	-2.68746484
H	9.76645275	-7.62754090	0.87411926	H	7.94005948	-7.95352015	-3.60159234
H	12.04161834	-6.59189469	0.87548822	H	8.08216512	-6.56062989	-4.65290202
H	12.04179629	-6.58942909	-0.87512120	H	5.78322997	-7.11985037	-2.72137267
H	11.84813865	-9.08176036	-0.87894507	H	5.75267687	-7.31383385	-4.45337569
H	11.84763149	-9.08451547	0.87178724	H	6.06792970	-4.87551395	-4.76645269
H	14.12122816	-8.04573004	0.87442510	H	6.18199009	-4.64359435	-3.03595352
H	14.12178995	-8.04164168	-0.87613077	H	3.87653744	-5.53117337	-2.74381982
H	13.93375509	-10.53425752	-0.88256775	H	3.73086995	-5.73527183	-4.47594680

H	13.93207951	-10.53886546	0.86821379	H	4.05807823	-3.28106391	-4.79783113
H	16.20269419	-9.49382236	0.87357626	H	4.23154372	-3.07309180	-3.06784116
H	16.20466056	-9.48788909	-0.87692452	H	1.91425813	-3.94200615	-2.72849232
H	16.02604796	-11.98094501	-0.88655288	H	1.74056578	-4.16099917	-4.45593457
H	16.02130412	-11.98779221	0.86427258	H	2.08240586	-1.69982733	-4.79268639
H	18.28745714	-10.93331863	0.87508452	H	2.21856274	-1.48992556	-3.05982689
H	18.29298809	-10.92571627	-0.87537734	H	-0.10045664	-2.38096759	-2.77169484
H	18.12757344	-13.41923208	-0.88833420	H	-0.23391576	-2.59172154	-4.50372774
H	18.11634149	-13.42844248	0.86247197	H	0.08894578	-0.13439914	-4.82924687
H	20.37688371	-12.36170745	0.88295658	H	0.21924590	0.07766538	-3.09633616
H	20.38933765	-12.35333314	-0.86748806	H	-2.23277114	-1.02670455	-4.53941564
H	20.21712443	-14.85805702	0.86785260	H	-2.09863957	-0.80221040	-2.80889488
H	20.23778552	-14.84719349	-0.88281155	H	-0.48705448	1.56619442	0.64999330
H	22.47319129	-13.78260301	0.90186685	H	-0.63164736	1.34945468	-1.08066468
H	22.49528132	-13.77208231	-0.84856593	H	-1.91862942	1.41916964	-4.89042921
H	22.31895021	-16.27970497	0.88227786	H	-1.75672796	1.65352343	-3.16502422
H	22.35044679	-16.26590557	-0.86821299	H	-2.66087184	0.39803821	0.98735591
H	24.57343736	-15.20273562	0.93323296	H	-2.80468136	0.15149734	-0.73794762
H	24.60617200	-15.18465158	-0.81681301	H	-2.70845954	2.88817710	0.63995006
H	24.42896642	-17.69590510	0.90444234	H	-2.85791312	2.63693260	-1.09838451
H	24.46214463	-17.67793489	-0.84467537	H	-4.15374026	2.12274700	-0.02022124
H	26.70740219	-16.63632091	0.96491599	H	-4.26334847	0.57254897	-4.55986816
H	26.73498038	-18.15460912	0.06777430	H	-4.09924535	0.81408020	-2.82147640
H	26.74060499	-16.61717066	-0.79647699	H	-4.07368414	2.20070661	-3.90895484

**$C_{28}H_{58}$ Linear Conformer**				**$C_{28}H_{58}$ Hairpin Conformer**			
86				86			
-29962.578265399 eV				-29962.881075482 eV			
C	-0.29812101	0.21820528	-0.00008181	C	-0.17938464	-0.46014347	-0.01285482
C	1.21944770	0.18641855	0.00006400	C	1.33317128	-0.41371639	-0.13516542
C	1.78326461	-1.22412209	0.00009947	C	1.99379516	-1.77563382	-0.01790008
C	3.30092746	-1.27060041	0.00030858	C	3.50657734	-1.72172305	-0.13355556
C	3.86178511	-2.68180800	0.00027872	C	4.17774164	-3.07539738	0.01523493
C	5.37951299	-2.72956006	0.00056975	C	5.69074958	-3.01013720	-0.09598369
C	5.93996688	-4.14086363	0.00031476	C	6.36574846	-4.36524758	0.01891742
C	7.45771393	-4.18825744	0.00065924	C	7.87836461	-4.29122094	-0.11521861
C	8.01879929	-5.59924327	-0.00011265	C	8.58426564	-5.64324639	-0.07832531
C	9.53652690	-5.64588138	0.00023056	C	8.15528701	-6.61446564	-1.17243608
C	10.09822598	-7.05658693	-0.00144064	C	8.48569236	-6.14704504	-2.58396466
C	11.61590720	-7.10230657	-0.00104194	C	7.74294455	-6.90277760	-3.68010989
C	12.17886830	-8.51248237	-0.00389953	C	6.22746450	-6.72845604	-3.65821942
C	13.69656842	-8.55596509	-0.00296029	C	5.75541437	-5.29089333	-3.80666080
C	14.26252404	-9.96492477	-0.00712112	C	4.24737541	-5.14611242	-3.70670157
C	15.78034270	-10.00407204	-0.00467463	C	3.76668090	-3.71114146	-3.83175855
C	16.35136587	-11.41099689	-0.00985702	C	2.26105344	-3.56927077	-3.69770767
C	17.86936956	-11.44368795	-0.00423687	C	1.76989685	-2.13859370	-3.82713572
C	18.44738556	-12.84779704	-0.00975653	C	0.26063771	-2.00973795	-3.72465253
C	19.96557928	-12.87211466	0.00141452	C	-0.23742666	-0.58204194	-3.86099261
C	20.55214180	-14.27270496	-0.00321367	C	-0.83616045	0.90421044	-0.12320881
C	22.07048316	-14.28759500	0.01645989	C	-1.74755090	-0.45780239	-3.76566509
C	22.66518756	-15.68468343	0.01406277	C	-2.24937331	0.96718586	-3.91721015
C	24.18350595	-15.69267509	0.04389240	C	-2.34768366	0.86227640	0.01422201
C	24.78242927	-17.08816191	0.04291694	C	-3.75827426	1.09251751	-3.79779782
C	26.30024230	-17.09265006	0.08270961	C	-3.00485470	2.22488471	-0.12052749
C	26.90450759	-18.48626103	0.07966198	C	-4.51515948	2.17619993	0.02101630
C	28.42186848	-18.47394714	0.12144685	C	-4.25330581	2.51876059	-3.95342722
H	-0.68033220	1.24052438	-0.00011579	H	-0.45090355	-0.91651632	0.94641523
H	-0.70134447	-0.28733793	0.88076818	H	-0.58590246	-1.12096219	-0.78713361

H	-0.70117721	-0.28733416	-0.88101056
H	1.59941748	0.72519124	-0.87463965
H	1.59924734	0.72516829	0.87485566
H	1.40378926	-1.76463330	-0.87527227
H	1.40354991	-1.76468591	0.87533474
H	3.67991569	-0.73015672	0.87579984
H	3.68016188	-0.73003075	-0.87499854
H	3.48255434	-3.22207600	-0.87518835
H	3.48220954	-3.22226031	0.87548209
H	5.75875980	-2.18944852	0.87613245
H	5.75910094	-2.18902740	-0.87458586
H	5.56089835	-4.68103340	-0.87529576
H	5.56046545	-4.68155405	0.87541532
H	7.83672723	-3.64828852	0.87641762
H	7.83713238	-3.64723429	-0.87427384
H	7.64005873	-6.13927640	-0.87594880
H	7.63959058	-6.14048366	0.87477404
H	9.91532472	-5.10628889	0.87632255
H	9.91574966	-5.10413415	-0.87434740
H	9.71978210	-7.59619952	-0.87766424
H	9.71922364	-7.59858194	0.87306837
H	11.99428284	-6.56317805	0.87552400
H	11.99482219	-6.55951131	-0.87510953
H	11.80133781	-9.05163658	-0.88079780
H	11.80016509	-9.05572564	0.86996459
H	14.07359693	-8.01708874	0.87434766
H	14.07487467	-8.01145218	-0.87622734
H	13.88716192	-10.50383819	-0.88509195
H	13.88432173	-10.51016190	0.86571484
H	16.15464142	-9.46500113	0.87368928
H	16.15782795	-9.45719015	-0.87681851
H	15.97988256	-11.95003014	-0.88939291
H	15.97355030	-11.95893007	0.86145239
H	18.23920686	-10.90397500	0.87562659
H	18.24627620	-10.89391429	-0.87481076
H	18.08182420	-13.38734092	-0.89144911
H	18.06938950	-13.39901806	0.85939549
H	20.32890578	-12.33124477	0.88327538
H	20.34262698	-12.31918730	-0.86709171
H	20.17283575	-14.82763331	0.86300371
H	20.19483539	-14.81316003	-0.88772856
H	22.42537832	-13.74523592	0.90082550
H	22.44899763	-13.73162625	-0.84946088
H	22.28317997	-16.24276979	0.87705531
H	22.31677130	-16.22608757	-0.87340853
H	24.53047690	-15.15068851	0.93160729
H	24.56578701	-15.13392609	-0.81855184
H	24.39578623	-17.64885559	0.90217111
H	24.44119422	-17.62850674	-0.84800432
H	26.64093021	-16.55372524	0.97489492
H	26.68749562	-16.52946951	-0.77479216
H	26.51682805	-19.04801696	0.93633828
H	26.56473850	-19.02343879	-0.81237913
H	28.78571802	-17.96997425	1.02014929
H	28.83383450	-19.48464636	0.11778587
H	28.83390110	-17.94400819	-0.74079371
H	1.60650685	0.04034562	-1.09530282
H	1.73803444	0.25165586	0.63664160
H	1.59363258	-2.44181541	-0.79102837
H	1.72005501	-2.22875906	0.94225298
H	3.90266598	-1.03662891	0.62547024
H	3.78135523	-1.28645458	-1.10219953
H	3.78755496	-3.76013778	-0.74731477
H	3.90275794	-3.51250300	0.98236660
H	6.08370090	-2.33637060	0.67455979
H	5.96191856	-2.55640823	-1.05744423
H	5.95282431	-5.02382162	-0.75177282
H	6.10691498	-4.82606963	0.97989273
H	8.27602687	-3.66499132	0.69040101
H	8.12755382	-3.76602487	-1.04340067
H	8.40419397	-6.10873551	0.89666467
H	9.66563107	-5.48032877	-0.14224984
H	7.08146824	-6.79523833	-1.08197593
H	8.63128216	-7.58499420	-0.99874721
H	9.56390988	-6.24408612	-2.74736358
H	8.26991160	-5.08047282	-2.68300304
H	7.97800845	-7.97021164	-3.60715290
H	8.12182897	-6.57404124	-4.65381042
H	5.81811088	-7.14295495	-2.73081815
H	5.79281390	-7.32983697	-4.46367286
H	6.10165379	-4.89064902	-4.76725815
H	6.21494144	-4.66394465	-3.03597874
H	3.91216779	-5.55570282	-2.74562104
H	3.76654484	-5.75962379	-4.47775244
H	4.08494953	-3.30401062	-4.79869733
H	4.25886775	-3.09629751	-3.06866266
H	1.94441472	-3.97560936	-2.72927488
H	1.77059320	-4.18965153	-4.45727174
H	2.10355775	-1.72673515	-4.78692448
H	2.24105958	-1.52112171	-3.05357550
H	-0.07402679	-2.42144314	-2.76483747
H	-0.20807774	-2.63141902	-4.49675399
H	0.10017699	-0.17116257	-4.81984715
H	0.22628184	0.03955525	-3.08637037
H	-2.21241051	-1.08859668	-4.53267743
H	-2.08326564	-0.85982509	-2.80241267
H	-0.42144797	1.56938971	0.64350812
H	-0.57219445	1.35561686	-1.08692109
H	-1.92836202	1.36379511	-4.88784622
H	-1.77381095	1.60227134	-3.16059010
H	-2.61196203	0.42678116	0.98529391
H	-2.76262968	0.18547881	-0.74190677
H	-2.58647972	2.90179826	0.63218916
H	-2.74014297	2.65456801	-1.09241734
H	-4.23274514	0.45420855	-4.55097032
H	-4.07371863	0.69760657	-2.82630096
H	-4.80566965	1.78691920	0.99989270
H	-4.96209139	3.16595261	-0.08869284
H	-4.96154784	1.52674728	-0.73585568
H	-3.98481661	2.92319428	-4.93244763
H	-5.33850920	2.58149451	-3.85449667
H	-3.81449525	3.17340188	-3.19659154

References

(1) Paukku, Y.; Yang, K. R.; Varga, Z.; Truhlar, D. G. Global ab initio ground-state potential energy surface of N_4. *J. Chem. Phys.* **2013**, *139*, 044309.

(2) Conte, R.; Houston, P. L.; Bowman, J. M. Communication: A benchmark-quality, full-dimensional ab initio potential energy surface for Ar-HOCO. *J. Chem. Phys.* **2014**, *140*, 151101.
