# Supplementary material for: “Gold-Standard” Δ‑Machine Learned Transferable Potential for Linear Alkanes
Source: J Phys Chem Lett. 2025 Nov 20;16(48):12393–400. doi: 10.1021/acs.jpclett.5c02946 (PMC12683630; doi:10.1021/acs.jpclett.5c02946)
Supplement: Supplementary file 2 [file jz5c02946_si_002.pdf]

jz-2025-02946c.R1

Name: Peer Review Information for ""Gold-Standard" Δ -Machine Learned and ΔTransferable Potential for Linear Alkanes"

First Round of Reviewer Comments

Reviewer: 1

Comments to the Author

This manuscript reports the combination of the many-body permutationally invariant polynomial (MB-PIP) framework with the Δ-machine learning (Δ-ML) approach to elevate two DFT-based potentials to CCSD(T) accuracy for alkanes. The authors have previously demonstrated the applicability of their MB-PIP approach for alkanes in a series of publications, and they have also employed Δ-ML strategies to construct CCSD(T)-level PESs for various gas-phase molecules. Here, the integration of these two schemes offers a reasonable route toward transferable and accurate potentials for larger alkane systems. The presentation is generally clear. However, I have several comments that should be addressed before I can recommend publication:

(1) I noticed some of the authors recently introduced a different approach, MB-PIPNet (Nat. Comput. Sci. 2025, 5, 418–426). What are the key differences between MB-PIP and MB-PIPNet? Clarifying this distinction is important to avoid confusion in the community.

(2) A related question concerns the distinction between the MB-PIP method and popular atomistic machine-learning potentials based on atomic energy decomposition. What are the advantages of MB-PIP relative to such approaches? Have the authors performed comparative benchmarks, particularly in terms of accuracy and computational efficiency?

(3) Analogous to the relationships among PIP, PIP-NN and FI-NN, it seems that neural networks incorporating PIP or FI terms could also be used to fit many-body atomic

interactions. Could the authors comment on the feasibility or advantages of such extensions?

(4) The current manuscript contains a detailed review of the MB-PIP and Δ-ML approaches together with extensive computational details. I suggest that some of these technical details be moved to the Supporting Information to improve focus and readability for a Letter.

(5) On Page 9, the reported fitting errors for the Δ-ML model are very small (12 and 7 cm^{-1}). Given that only 4514 configurations were included, are there any overfitting issues?

(6) Some text need careful proofread (line 23, Page 3the full name of DLPNO is domain-based local pair natural orbital ; lines 45–48, Page 3; etc.)

Reviewer: 2

Comments to the Author

The authors present a Delta-ML correction applied to an existing many-body permutationally invariant polynomial (MB-PIP) potential that previously lacked dispersion. The Delta correction is trained on PNO-LCCSD(T)-F12 reference energies; a parallel MB-PIP trained on PBE0+MBD is also presented for comparison. The corrected potentials are applied to medium-sized linear alkanes and used to compute power spectra from molecular dynamics (MD) simulations.

If the major issues below - particularly those on reproducibility, clarity, and evidence for the claimed scientific advance - are addressed, the manuscript could be publishable. However, given the limited novelty relative to prior literature on folded vs. unfolded alkane conformations (see https://onlinelibrary.wiley.com/doi/10.1002/anie.201202894, https://pubs.acs.org/doi/full/10.1021/acs.jpca.2c02439), I believe the paper in its current form is not well suited for J. Phys. Chem. Lett. and would be more appropriate for a journal

with a broader focus. The authors should also better frame their motivation: if the stated motivation is "missing dispersion in our earlier MB-PIP," they must justify why a coupled-cluster correction (1) is the appropriate remedy despite its expense, (2) is more appropriate to established dispersion corrections (e.g., DFT-D3/D4, VV10), and (3) does not simply conflate method and basis-set errors with the physical dispersion term. (4) A justification on why disperision interactions were not accounted for in the previous work despite cheap and broadly applicable corrections being availible for quite some time now.

1 Adequacy / Suitability for JPC Letters

- Main point: The physical problem (folded vs. unfolded alkane conformations and the role of dispersion) is important but has been extensively studied; the manuscript does not convincingly demonstrate a frontier advance in method or understanding that warrants JPC Letters' scope. The authors should either (a) significantly strengthen the novelty/impact claims (e.g., new physical insight enabled uniquely by the Delta-ML CC correction), or (b) consider submitting to a specialty journal focused on computational chemistry or molecular modeling.

2 Reproducibility (important, move this to the paper's "Computational Details" & SI; make public code/data)

This is a critical deficiency. In its present state the manuscript cannot be accepted because it lacks the information and materials needed to reproduce results.

What must be provided (minimum, to accompany the revised manuscript / SI / repository):

1. Complete computational details in the manuscript and SI:

- Levels of theory and functionals (e.g., PBE0), full basis sets for all calculations (DFT and CC), use of F12, auxiliary basis sets, and any RI/auxiliary approximations.

- Precise definition of the Δ-ML training target (reference energies used, whether energies are relative to a chosen conformer, counterpoise corrections if used, etc.).

- Description of dataset selection: Diversity metrics.

2. MD simulation details: thermostat/barostat type, target temperature(s), pressure (if NPT), timestep, total simulation length, ensemble, integrator, periodic boundary conditions, cutoffs, electrostatic handling, software name and version, and any force-field/ML-potential implementation specifics.

3. ML training details: model architecture, hyperparameters, training/validation/test splits, loss function, training convergence criteria, random seeds, software packages and versions, and the exact training script or a clear recipe.

4. Reference data & inputs: Provide the CC reference energies (or a representative subset) in the SI; provide input files (or scripts) for key electronic structure calculations.

5. Code & models: release the MB-PIP code and/or a compiled binary and the Delta-ML model weights in a public repository (e.g., GitHub, with DOI via Zenodo). If licensing or IP is a concern, provide clear instructions for obtaining the binaries and an archival copy of the training data.

6. Geometries: provide XYZ (or other standard) files for all structures mentioned explicitly in the text (hairpin, linear conformers, representative MD snapshots), ideally in the repository.

7. Units & reference: report all energies in kcal mol^-1 (and optionally kJ mol^-1) with clear reference states.

3 Citations

- The manuscript frequently omits citations where they are expected (especially in the Introduction). Add some recent reviews on alkane conformational behavior and dispersion effects so readers (especially early-career researchers) can quickly access background

literature. Cite competing approaches for including dispersion (e.g. Grimmes DFT-D and VV10 paper/review article) when motivating the Delta-ML CC approach.

4 Clarity, language, and presentation

- Many sentences are awkwardly ordered, some figure captions are not self-contained, and there are some spelling and grammatical errors. The authors should thoroughly proofread the manuscript. Captions must explain symbols and main messages independently of the main text.

5 Detailed, numbered technical criticisms

----

1. Page 3, line 35: Clarify the sentence that refers to "the change" - specify for which alkane the change happens.

2. Page 3, line 54: Move the sentence that references a figure to the location where the two conformations are first introduced so the figure and text align.

3. Page 4, figure caption: Improve the caption by explaining the origin of displayed structures (e.g., optimized at X level, extracted from MD at Y), how they were selected, and any color/label conventions.

4. Page 5, line 15 (folding landscape): Define what is meant by "folding landscape" (potential of mean force? energy landscape?).

5. Page 5, line 9 (B3LYP comment): "250,000 calculations on one alkane with B3LYP seems excessive." The authors should justify the sampling strategy, report the exact functional and basis set, and explain computational cost vs. benefit.

6. Page 6, Formula (1): Define every variable and symbol immediately after the equation. Provide a short sentence interpreting the equation in words.

7. Page 6, line 11: Reword the misleading sentence so it precisely states the intended idea.

8. Page 9, line 19: State the basis set(s) used for the CC calculations (including density fitting and RI basis sets). Was the CABS singles correction applied?

9. Page 9, line 31 (MD details missing): Provide full MD protocol: program and version, thermostat/barostat algorithms, target T and P, timestep, cutoffs, simulation lengths, how the MB-PIP/Delta-ML potentials were interfaced (plug-in or custom code), and whether trajectories were equilibrated.

10. Page 9, line 40 (configuration diversity): Explain how configurations for CC calculations were selected, and provide metrics for diversity (e.g., clustering counts, RMSD ranges, energy ranges). If selection was biased, justify it.

11. Page 10, line 15 (transferability): If transferability is claimed, show transferability also for the PBE0+MBD PES or clearly justify why not.

12. Page 10, line 23 (structure origins): State how the hairpin and linear structures were obtained (optimization level, initial guesses). Provide XYZ files in the public repository.

13. Page 10, line 35 (naming conventions): Current labels such as "B3LYP PES" vs. "DeltaB3LYP PES" are confusing. Use systematic nomenclature (e.g., MB-PIP_{B3LYP}, MB-

PIP_{B3LYP+DeltaCC}) or include theory as subscripts/superscripts to avoid reader confusion. Define names in a single table.

14. Figure 2 caption: Make the caption self-contained

15. Table 1: Move table to the SI; keep only summary values in the main text unless the table is central to the argument.

16. Page 13, line 52: Name the electronic-structure / MD program(s) used (including version numbers).

17. Page 14, line 5 (spectra discussion): The comparison of harmonic normal-mode spectra and MD-derived spectra is weak. Provide a clearer analysis: assign peaks, compare to experiment if available, and quantify agreement (peak positions, intensities) rather than qualitative similarity.

18. Page 17, line 13 (conclusions on dispersion): I agree with the claim that dispersion is of crucial importance howver this should be made more clearer in the main text, when discussing the energy differences between hairpin and linear minima in comparison to CC reference data. Furthermore, this is a fact that has been discussed extensively in various papers over the last 15 years, particularly in the context of alkane folding.

Author's Response to Peer Review Comments:

Responses to reviews of “Gold-Standard” Δ-Machine Learned and Transferable Potential for Linear Alkanes

Chen Qu,[*,†] Apurba Nandi,[*,‡] Paul L. Houston,[§] Qi Yu,[‖] Riccardo Conte,[⊥] and

Joel M. Bowman*,#

†*Independent Researcher, Toronto, Ontario M9B0E3, Canada*

‡*Department of Physics and Materials Science, University of Luxembourg, L-1511, Luxembourg City, Luxembourg.*

¶*Dipartimento di Chimica, Università degli Studi di Milano, via Golgi 19, 20133 Milano, Italy*

§*Department of Chemistry and Chemical Biology, Cornell University, Ithaca, New York 14853, USA and Department of Chemistry and Biochemistry, Georgia Institute of Technology, Atlanta, Georgia 30332, USA*

‖*Department of Chemistry, Fudan University, Shanghai, 200438, P. R. China*

⊥*Dipartimento di Chimica, Università Degli Studi di Milano, via Golgi 19, 20133 Milano, Italy*

#*Department of Chemistry and Cherry L. Emerson Center for Scientific Computation, Emory University, Atlanta, Georgia 30322, USA.*

E-mail: szquchen@gmail.com; apurba.nandi@uni.lu; jmbowma@emory.edu

We are gratified that both reviewers rated the research reported "high" in both significance and novelty. We are also grateful for the helpful comments which we have addressed in the revision. Details are given below. Our responses are in blue font and the changes in the marked-up pdf are given in red font

Reviewer: 1

Recommendation: This paper may be publishable, but major revision is needed; I would like to be invited to review any future revision.

Comments: This manuscript reports the combination of the many-body permutationally invariant polynomial (MB-PIP) framework with the Δ-machine learning (Δ-ML) approach to elevate two DFT-based potentials to CCSD(T) accuracy for alkanes. The authors have previously

demonstrated the applicability of their MB-PIP approach for alkanes in a series of publications, and they have also employed Δ-ML strategies to construct CCSD(T)-level PESs for various gas-phase molecules. Here, the integration of these two schemes offers a reasonable route toward transferable and accurate potentials for larger alkane systems. The presentation is generally clear. However, I have several comments that should be addressed before I can recommend publication:

(1) I noticed some of the authors recently introduced a different approach, MB-PIPNet (Nat. Comput. Sci. 2025, 5, 418–426). What are the key differences between MB-PIP and MB-PIPNet? Clarifying this distinction is important to avoid confusion in the community.

Thanks for this comment and we appreciate that the reviewer is aware of the MB-PIPNet method. In short, the current MB-PIP approach is designed to employ PIPs to describe atomic many-body terms, such as atomic 3-body and 4-body interactions. A linear least squares fit is used to obtain the model with all the PIP bases. The MB-PIPNet approach is a conceptually different one which decomposes the total energy into the sum of effective monomeric energies. Each monomer is described by a neural network to obtain its effective energy. We plan to extend the MB-PIPNet approach to alkanes in the near future, along with other ML methods. This would be a major paper of course and beyond the scope of the present letter. However, we gladly mention this method with the following new text It should also be noted that recently, a conceptually different approach, MB-PIPNet, was introduced by some of us, which decomposes the total energy of the system into sum of effective monomeric energies using only monomeric 1-b and 2-b descriptors.[1] Extension of the MB-PIPNet framework to systems such as alkanes is under investigations and will be reported in the future.

(2) A related question concerns the distinction between the MB-PIP method and popular atomistic machine-learning potentials based on atomic energy decomposition. What are the advantages of MB-PIP relative to such approaches? Have the authors performed comparative benchmarks, particularly in terms of accuracy and computational efficiency?

This is a reasonable question and certainly something for future work. We have done some preliminary timing tests using MACE-OFF and indeed, MB-PIP is much faster than MACE-OFF. We prefer not to go into much detail since doing a careful comparison in terms of accuracy (vis-a-vis the CC results) and timing is beyond the scope of the paper. However, we have added text near the end of the paper. Our expectation is that these methods will run slower than the current one, owing to the much larger number of learned parameters and complexity of these approaches. However, at this point this is speculation.

(3) Analogous to the relationships among PIP, PIP-NN and FI-NN, it seems that neural networks incorporating PIP or FI terms could also be used to fit many-body atomic interactions. Could the authors comment on the feasibility or advantages of such extensions?

Yes, that is correct and an important point to include. We have added text to reflect this. We do note that the many-body expansion up to the 4-body term effectively includes diatomic, triatomic, and tetraatomic "molecules." These are quite small, so a variety of methods can be applied to them, including GPR, provided the approach is permutationally invariant. So, PIP-GPR and FI-GPR are also mentioned.

We have added the following text:

> Note, the MB-PIP approach with up to 4-body terms involves diatomic, triatomic, and tetraatomic "molecules", a variety of permutationally invariant methods such as PIP-NN/FINN and PIP-GPR/FI-GPR can also be employed.

(4) The current manuscript contains a detailed review of the MB-PIP and Δ-ML approaches together with extensive computational details. I suggest that some of these technical details be moved to the Supporting Information to improve focus and readability for a Letter.

We have done that. Thanks for the suggestion.

(5) On Page 9, the reported fitting errors for the Δ-ML model are very small (12 and 7 cm^{-1}). Given that only 4514 configurations were included, are there any overfitting issues?

Thanks for the comment — we should have addressed this point. The small MAE is a reflection of the fact that we are fitting a difference energy, which is much smaller in magnitude than the total energy itself. Consequently, the MAE is expected to be correspondingly smaller than that obtained when fitting the total energy. In this case, the ratio between the energy and the difference energy is about 10, which indeed matches the factor difference between the respective MAEs. We have expanded the text in the section discussing the MAEs to clarify these points. These small fitting errors are not surprising and do not indicate overfitting because the range of the energy difference is much smaller, specifically, the energy differences between PNO-LCCSD(T)-F12 and B3LYP span a range of 12 966 cm^{-1}, and the differences between PNO-LCCSD(T)-F12 and PBE0+MBD span only 3904 cm^{-1}.

(6) Some text need careful proofread (line 23, Page 3the full name of DLPNO is domainbased local pair natural orbital ; lines 45–48, Page 3; etc.)

There are two CCSD(T) methods mentioned in the paper. One is DLPNO-CCSD(T) and the other, the one that we use, is PNO-LCCSD(T)-F12. We have checked and made any necessary corrections to use proper notation in the revision. Also, we have made stylistic changes in response to the comments of Reviewer 2.

Additional Questions: Urgency: Top 10

Significance: High
Novelty: High

Scholarly Presentation: Top 10

Is the paper likely to interest a substantial number of physical chemists, not just specialists working in the authors' area of research?: Yes

Reviewer: 2

Recommendation: This paper may be publishable, but major revision is needed; I would like to be invited to review any future revision.

Comments: The authors present a Delta-ML correction applied to an existing many-body permutationally invariant polynomial (MB-PIP) potential that previously lacked dispersion.

The Delta correction is trained on PNO-LCCSD(T)-F12 reference energies; a parallel MBPIP trained on PBE0+MBD is also presented for comparison. The corrected potentials are applied to medium-sized linear alkanes and used to compute power spectra from molecular dynamics (MD) simulations.

I believe the paper in its current form is not well suited for J. Phys. Chem. Lett. and would be more appropriate for a journal with a broader focus. The authors should also better frame their motivation: if the stated motivation is "missing dispersion in our earlier MB-PIP," they must justify why a coupled-cluster correction (1) is the appropriate remedy despite its expense, (2) is more appropriate to established dispersion corrections (e.g., DFT-D3/D4, VV10), and (3) does not simply conflate method and basis-set errors with the physical dispersion term. (4) A justification on why disperision interactions were not accounted for in the previous work despite cheap and broadly applicable corrections being availible for quite some time now.

Our responses to the four points raised above are as follows. First, points (1) - (3). It seems we were not clear enough about the novelty and goal of the present work. So to be clear, we are *not* presenting another *direct* calculation of the linear and hairpin minima of alkanes. We are reporting a transferable ML potential for linear alkanes that is "Δ-corrected", using one of the best benchmark-tested CCSD(T) methods that can be applied for training.

We are *using* these minima as tests of the new Δ-corrected MLPs. We go on to present power spectra using one DFT and then the Δ-corrected DFT MLP. These were not reported previously in any of the fine electronic structure papers that we refer to in the Introduction. Next, why did we choose "CCSD(T)" instead of one of the dispersion corrections to DFT, as mentioned in (2). Very simply, our goal is to demonstrate that we can "Δ-correct" to the CCSD(T) level for a transferable MLP of alkanes. We have been doing this for a few years for molecules such as acetyl acetone, tropolone, and ethanol (and are known for this.) So, we are a bit surprised that the reviewer

doesn't appreciate this, rather than using one or another dispersion correction to DFT. We have added text in the revision to make this clearer and we also note at the end of the paper that various dispersion corrected DFT methods can now be tested against the results we show for the large range of alkanes and of course the new database of 4515 PNO-LCCSD(T)-F12 energies for $C_{14}H_{30}$. These changes are made in red font in various places in the marked-up pdf. We thank the reviewer for raising these points as they indicate we were not clear enough about the novelty and significance of the work. About point (4): We goofed. However, correcting this at the PNO-LCCSD(T)-F12 level allows us to see the large effect of dispersion (and of course post-DFT treatment of the electronic structure). The new PBE+MBD MLP does provide a nice example of the success of a dispersion-corrected DFT method. However, as we showed, even that method can be
nicely Δ-corrected.

1 Adequacy / Suitability for JPC Letters ————————————————- - Main point: The physical problem (folded vs. unfolded alkane conformations and the role of dispersion) is important but has been extensively studied; the manuscript does not convincingly demonstrate a frontier advance in method or understanding that warrants JPC Letters' scope. The authors should either (a) significantly strengthen the novelty/impact claims (e.g., new physical insight enabled uniquely by the Delta-ML CC correction), or (b) consider submitting to a specialty journal focused on computational chemistry or molecular modeling. We have addressed these concerns above. The work is both novel and significant to developers and people following advances in MLPs, as well as the large community specifically interested in alkanes. We expect these MLPs will find wide usage, as have many of our PIP-based MLPs.

2 Reproducibility (important, move this to the paper's "Computational Details" SI; make public code/data) ———————————————————————————— This is a

critical deficiency. In its present state the manuscript cannot be accepted because it lacks the information and materials needed to reproduce results. Revisions have been made in response; details are below.

What must be provided (minimum, to accompany the revised manuscript / SI / repository):

1. Complete computational details in the manuscript and SI: - Levels of theory andfunctionals (e.g., PBE0), full basis sets for all calculations (DFT and CC), use of F12, auxiliary basis sets, and any RI/auxiliary approximations. - Precise definition of the -ML training target (reference energies used, whether energies are relative to a chosen conformer, counterpoise corrections if used, etc.). - Description of dataset selection: Diversity metrics. There are various points raised here which we did address and have also expanded on revision in red font. Specifically we give all the relevant details of the PBE0+MBD, B3LYP and PNOLCCSD(T)-F12 calculations including the software used. We are clear about the reference energies used for the -ML training. We are not using any counterpoise corrections etc. If we had used any such corrections, we would have mentioned that.

2. MD simulation details: thermostat/barostat type, target temperature(s), pressure (ifNPT), timestep, total simulation length, ensemble, integrator, periodic boundary conditions, cutoffs, electrostatic handling, software name and version, and any force-field/ML-potential implementation specifics.These details are given in the revised manuscript. Specifically, the classical trajectories adopted a step size of 5.0 a.u. (0.121 fs); each trajectory were equilibrated for 10,000 steps (1.2 ps), and continued for another 22,500 steps (2.7 ps). The final spectra are averages for five trajectories at each temperature and potential. Here we considered both the linear and hairpin minima. The trajectories were run using the NVE protocol and for zero total angular momentum using our in-house software.

3. ML training details: model architecture, hyperparameters, training/validation/testsplits, loss function, training convergence criteria, random seeds, software packages and versions, and

the exact training script or a clear recipe.These details were given in the manuscript and in references cited. We have moved details to the Supporting Information in accord with the suggestion of reviewer 1. The software including details on the linear least-squares software has been given by us in technical papers. We point the reader to ref. 42 for details. We see no reason to repeat those details here.

4. Reference data and inputs: Provide the CC reference energies (or a representativesubset) in the SI; provide input files (or scripts) for key electronic structure calculations.

These data are available as we wrote in the manuscript in the Data Availability section.

We much prefer to have the data on the the gihub repository and don't wish to include a "representative subset" in the SI since we don't know what objective that would satisfy. Also we noted that the PES is available upon request to the authors. So no changes are made to the revised manuscript related to this.

5. Code and models: release the MB-PIP code and/or a compiled binary and the DeltaML model weights in a public repository (e.g., GitHub, with DOI via Zenodo). If licensing or IP is a concern, provide clear instructions for obtaining the binaries and an archival copy of the training data. As noted above, we include information on how to obtain the code in the Data Availability section. We don't revise this availablity option, which is our decision to make. We have made our potential codes available in the way we state for more than
twenty years.

6. Geometries: provide XYZ (or other standard) files for all structures mentioned explicitly in the text (hairpin, linear conformers, representative MD snapshots), ideally in the repository. The coordinates of all minima are now included as part of the Supporting Information. We don't see the point in including coordinates of some of the thousands of "MD snapshots" and so we decline to do that.

7. Units and reference: report all energies in kcal mol^{-1} (and optionally kJ mol^{-1}) with clear reference states. In summary, we have addressed all of these points in the revision. We have

provided an SI with details of MLP parameters as well as moved Table 1 there. We also provided a file containing the coordinates of all minima. A github repository containing the 4515 CCSD(T) configurations and energies is given. We state the the MLPs are available upon request to the authors.

3 Citations ——————— - The manuscript frequently omits citations where they are expected (especially in the Introduction). Add some recent reviews on alkane conformational behavior and dispersion effects so readers (especially early-career researchers) can quickly access background literature. Cite competing approaches for including dispersion (e.g. Grimmes DFT-D and VV10 paper/review article) when motivating the Delta-ML CC approach. We did cite eleven papers dating from 1994 to 2023 relevant to alkane conformation. We have added a citation to another Grimme paper related to alkanes and dispersion corrections mentioned by the reviewer. *However*, we remind the reviewer that our paper is NOT focused on dispersion corrections to DFT and so an extensive review and discussion of those methods is tangential at best. Our paper is about Δ-correcting DFT MLPs using PNO-LCCSD(T)-F12 energies. Focusing on dispersion corrections to DFT would be an interesting study for a different paper, perhaps written by someone who is more experienced and invested in such corrections. We have added text at the end of the paper inviting such a study. Our relatively large dataset of PNO-LCCSD(T)-F12 energies provide benchmarks for various DFT+dispersion approaches and we have added text that effect at the end of the paper. These energies are available on a large github repository, as indicated in the paper. 4 Clarity, language, and presentation ———————————————— - Many sentences are awkwardly ordered, some figure captions are not self-contained, and there are some spelling and grammatical errors. The authors should thoroughly proofread the manuscript. Captions must explain symbols and main messages independently of the main text.

5 Detailed, numbered technical criticisms ————————————————

1. Page 3, line 35: Clarify the sentence that refers to “the change” - specify for whichalkane the change happens. The reviewer is referring to the change in the order of the minima and at

which alkane this occurs. We did indicate this when we discussed the Liakos and Neese paper. So we made no changes to the text.

2. Page 3, line 54: Move the sentence that references a figure to the location where thetwo conformations are first introduced so the figure and text align. Done

3. Page 4, figure caption: Improve the caption by explaining the origin of displayedstructures (e.g., optimized at X level, extracted from MD at Y), how they were selected, and any color/label conventions. We have added text indicating the paper where our work $C_{14}H_{30}$, the alkane shown, was first reported.

4. Page 5, line 15 (folding landscape): Define what is meant by “folding landscape” (potential of mean force? energy landscape?). We modified the text which now simply states the title of a paper which includes the term “energy landscape” recently published and cited.

5. Page 5, line 9 (B3LYP comment): “250,000 calculations on one alkane with B3LYPseems excessive.” The authors should justify the sampling strategy, report the exact functional and basis set, and explain computational cost vs. benefit.We added text about this dataset and then note that details have already been reported in ref.[2] The new text is ...that span a large energy range up to 230 kcal/mol, relative to the global minimum. Details of this extensive dataset are given in ref. 2. The energies and forces were obtained using the Gaussian 16 computational chemistry package.[3]

6. Page 6, Formula (1): Define every variable and symbol immediately after the equation.Provide a short sentence interpreting the equation in words. That expression is “generic” as we noted. We did give some specifics when we noted the application to water, where we defined the “body” and then provided four citations for details. We have added text stating that details of each term are found in those citations. When we get to the application to alkanes we do provide specifics on each term in that equation. So aside for adding the text noted above we have not made more revisions about this.

7. Page 6, line 11: Reword the misleading sentence so it precisely states the intendedidea. Not sure what is meant by “misleading”. Perhaps this is a language issue. We stated that the MB expansion is well-known for non-covalent interactions, which it is. And as stated above we gave the well-known case of water as an example. We are surprised that reviewer feels this needs more explanation. Again the all important details for our alkane work are given following that equation.

8. Page 9, line 19: State the basis set(s) used for the CC calculations (including densityfitting and RI basis sets). Was the CABS singles correction applied? We gave that information in the original paper, but have moved it up to the second paragraph of the revision. The text reads ...PNO-LCCSD(T)-F12b/AVTZ’ energies (where AVTZ’ means cc-pVTZ basis for H and aug-cc-pVTZ for C) (with a citation to the Werner-Hansen paper where this basis was used and described in great detail). We also expanded the acknowledgement to Andreas Hansen whose expert advice on using that basis we followed in doing the coupled cluster calculations. This is sufficient information for users of Molpro.

9. Page 9, line 31 (MD details missing): Provide full MD protocol: program and version,thermostat/barostat algorithms, target T and P, timestep, cutoffs, simulation lengths, how the MB-PIP/Delta-ML potentials were interfaced (plug-in or custom code), and whether trajectories were equilibrated. We have provide details of the MD calculations, in red font which were done with our own code (developed over more than 20 years) We stated that the MD calculations were “NVE", so readers will know that no thermostat or barstat is used in such calculations.

10. Page 9, line 40 (configuration diversity): Explain how configurations for CC calculations were selected, and provide metrics for diversity (e.g., clustering counts, RMSD ranges, energy ranges). If selection was biased, justify it. We now give details of how these configurations were obtained. The energies and coordinates are given in a github repository. So interested readers have access to these data.

11. Page 10, line 15 (transferability): If transferability is claimed, show transferabilityalso for the PBE0+MBD PES or clearly justify why not. We have added text to make it clear that training is done on $C_{14}H_{30}$ for the PBE0+MBD PES as it was previously for the B3LYP PES. So the results shown for all other alkanes in Figure 3 (and Figure 2) demonstrate the transferability of the PESs. The "claim" of transferability was verified in great detail in ref. 4 for the original B3LYP-based MLPs.

12. Page 10, line 23 (structure origins): State how the hairpin and linear structures wereobtained (optimization level, initial guesses). Provide XYZ files in the public repository. We stated in the manuscript "For consistency, we use stationary configurations for these alkanes, obtained from direct optimization of the relatively efficient and (as seen below) accurate PBE0+MBD method." So that information was given. We really don't see the need to give details about the optimization procedure as these are standard in the software we used and which is given in the revision in red font. We have provided the data requested about the minima in the new Supporting Information Section of the paper.

13. Page 10, line 35 (naming conventions): Current labels such as "B3LYP PES" vs. "DeltaB3LYP PES" are confusing. Use systematic nomenclature (e.g., MB-PIP B3LYP, MB-PIP B3LYP+DeltaCC) or include theory as subscripts/superscripts to avoid reader confusion. Define names in a single table. This is now actually done in response the point 14 where we define Δ-B3LYP. We prefer our notation to the one suggested. We just have the two DFT PESs and then the Δ-corrected ones. Confusion on the part the reader. However to be crystal clear, we have added text to indicate the when we refer to "PES" or "MLP" we mean "MB-PIP". This is indicated in red font

14. Figure 2 caption: Make the caption self-contained. Done and the revised caption is in red font.

15. Table 1: Move table to the SI; keep only summary values in the main text unless thetable is central to the argument. We have done that in the revision.

16. Page 13, line 52: Name the electronic-structure / MD program(s) used (includingversion numbers). We have added text and a citation about the package used to obtain the B3LYP/aVDZ

energies in the part of of the text where we do the same for the PBE0+MBD and CCSD(T) calculations. We added text that we use our own MD code to do the NVE trajectories. We have added text in ref font at the first mention of MD calculations to that effect. The software used to obtain the power spectra are cited already.

17. Page 14, line 5 (spectra discussion): The comparison of harmonic normal-mode spectra and MD-derived spectra is weak. Provide a clearer analysis: assign peaks, compare to experiment if available, and quantify agreement (peak positions, intensities) rather than qualitative similarity. First, the "assignment" of the many peaks is not an informative task for such a large molecule even at the harmonic level, where one could in principle examine each normal mode vector. We did note that the high frequency features are CH-stretches. Our goal was simply to indicate that the new MB-PIP PES can be used in the calculation of the power spectrum. We anticipate numerous applications of the new PES in future papers.

18. Page 17, line 13 (conclusions on dispersion): I agree with the claim that dispersionis of crucial importance however this should be made more clearer in the main text, when discussing the energy differences between hairpin and linear minima in comparison to CC reference data. Furthermore, this is a fact that has been discussed extensively in various papers over the last 15 years, particularly in the context of alkane folding. We agree that this is well known and and we are not adding much to this except perhaps by showing numerically the size of the effect for the difference in energies between linear and hairpin minima. We have text to this effect that starts with "We note that the importance..." when we conclude the discussion of Figure 2.

Additional Questions: Urgency: Moderate

Significance: High

Novelty: High

Scholarly Presentation: Moderate

Is the paper likely to interest a substantial number of physical chemists, not just specialists working in the authors' area of research?: Yes

References

(1) Yu, Q.; Ma, R.; Qu, C.; Conte, R.; Nandi, A.; Pandey, P.; Houston, P. L.; Zhang, D. H.; Bowman, J. M. Extending atomic decomposition and many-body representation with a chemistry-motivated approach to machine learning potentials. 2025.

(2) Qu, C.; Houston, P. L.; Allison, T.; Schneider, B. I.; Bowman, J. M. DFT-Based Permutationally Invariant Polynomial Potentials Capture the Twists and Turns of $C_{14}H_{30}$. *J. Chem. Theory Comput.* 2024, *20*, 9339–9353.

(3) Frisch, M. J. et al. Gaussian 16 Revision C.01. 2016; Gaussian Inc. Wallingford CT.

(4) Qu, C.; Houston, P. L.; Allison, T.; Bowman, J. M. Targeted Transferable Machine-Learned Potential for Linear Alkanes Trained on $C_{14}H_{30}$ and Tested for C_4H_{10} to $C_{30}H_{62}$. *J. Chem. Theory Comput.* 2025, *21*, 3552–3562.

jz-2025-02946c.R2

Name: Peer Review Information for ""Gold-Standard" Δ -Machine Learned and ΔTransferable Potential for Linear Alkanes"

Second Round of Reviewer Comments

Reviewer: 1

Comments to the Author

The authors have addressed my previous concerns properly. I recommend the manuscript be published in its current form.

Reviewer: 2

Comments to the Author

The manuscript has been improved from its original form by enhancing the computational details and by providing a supporting information section. It is now more comprehensible for the reader that a pre-trained potential computed at a "low" level of theory can be corrected using a small set of "high" level data. However, in my opinion. a minor revision is still necessary since the following comments from the first reviewing report were not addressed appropriately:

Motivation:

The authors remain unclear about their central goal. In the response they state: "Very simply, our goal is to demonstrate that we can 'Delta-correct' to the CCSD(T) level for a transferable MLP of alkanes." In the manuscript they write: "We note here and also in a very recent paper that dispersion is not accounted for in these straight B3LYP energies. So the central objective of the work here is to correct this in a single step by using Delta-ML at the CCSD(T) level." These two claims are inconsistent and must be reconciled.

Especially their claim that "However, the results in Figure 2 provide quantitative measure of dispersion over this large range of alkanes." should be carefully revised. The authors conflate method and basis-set errors with the physical dispersion term although this is for B3LYP mostly dispersion (but not exclusively). The authors should exchange their wording from the paper with the one provided in the response letter.

Citations:

The authors have not added enough citations, especially in the introduction (as previously commented). Specifically, the first two sentences of the introduction need additional and more appropriate citations. They read: "Hydrocarbons are important in fuels, plastics, and other industrial products. Consequently, there have been many studies, not only of their chemistry, but also of their physical and molecular properties." Citing Patterson alone is

insufficient here. The abbreviation "aVDZ" should be introduced (aug-cc-pVDZ) and appropriate citations for both the functional and the basis set should be provided.

For the phrase "We note here and also in a very recent paper" a citation is missing. Please add it.

Clarity:

As already mentioned in the first revision, sentences often wrongly ordered and the work is not self contained.

Especially the following phrases are hard to follow and need the suggested rewrite:

The sentence "We recently reported a transferable, many-body permutationally invariant polynomial (MB-PIP) potential for linear alkanes using roughly 253000 B3LYP electronic energies trained on C14 H30." needs rewriting because it reads as if B3LYP has been trained. I advise the authors to write the sentence like this: "We recently reported a transferable, many-body permutationally invariant polynomial (MB-PIP) potential for linear alkanes trained on roughly 253000 B3LYP/(insert basis set here) electronic energies of C14H30 conformations."

Also rewrite this sentence for clarity: "These focus on the minimum chain length where the transition from the linear minimum to the hairpin minimum occurs." I suggest: "These studies focus on identifying the shortest chain length at which the hairpin structure becomes more stable, i.e., energetically more favorable, than the linear structure."

For Formula (1) and (2) the authors must define every term. This has already been commented in the first revision. For example they should write that N refers to the number of atoms, V is a potential etc. This is currently not self contained. Otherwise they can mean anything and thus provides no worth to the manuscript.

Configuration diversity:

The authors have much improved by clarifying how the configurations for the coupled cluster calculations were obtained. However, still metrics for diversity have not been included in the revised manuscript. Figure 3 of the authors previous

paper(https://pubs.acs.org/doi/10.1021/acs.jctc.4c00932) is informative. I encourage the authors to present a similar figure for the distribution of coupled cluster energies.

Performance:

I agree with referee 1 that comparative benchmarks, particularly in terms of accuracy and computational efficiency are of high interest.

The authors should remove their speculative statements from the paper and rather provide some performance and timing comparisons in the supporting information.

Author's Response to Peer Review Comments:

Responses to reviews of "Gold-Standard" Δ-Machine Learned and Transferable Potential for Linear Alkanes

Chen Qu,[*,†] Apurba Nandi,[*,‡] Paul L. Houston,[§] Qi Yu,[k] Riccardo Conte,[⊥] and Joel M. Bowman[*,#]

†Independent Researcher, Toronto, Ontario M9B0E3, Canada

‡Department of Physics and Materials Science, University of Luxembourg, L-1511, Luxembourg City, Luxembourg.

¶Dipartimento di Chimica, Università degli Studi di Milano, via Golgi 19, 20133 Milano, Italy

§Department of Chemistry and Chemical Biology, Cornell University, Ithaca, New York 14853, USA and Department of Chemistry and Biochemistry, Georgia Institute of

Technology, Atlanta, Georgia 30332, USA

ₖ*Department of Chemistry, Fudan University, Shanghai, 200438, P. R. China*

⊥*Dipartimento di Chimica, Università Degli Studi di Milano, via Golgi 19, 20133 Milano, Italy*

#*Department of Chemistry and Cherry L. Emerson Center for Scientific Computation, Emory University, Atlanta, Georgia 30322, USA.*

E-mail: szquchen@gmail.com; apurba.nandi@uni.lu; jmbowma@emory.edu

Reviewer: 1

Recommendation: This paper represents a significant new contribution and should be published as is.

Comments: The authors have addressed my previous concerns properly. I recommend the manuscript be published in its current form.

Additional Questions: Urgency: High

Significance: High

Novelty: High

Scholarly Presentation: High

Is the paper likely to interest a substantial number of physical chemists, not just specialists working in the authors' area of research?: Yes

Reviewer: 2

Recommendation: This paper is publishable subject to minor revisions noted. Further review is not needed.

Comments: The manuscript has been improved from its original form by enhancing the computational details and by providing a supporting information section. It is now more comprehensible for the reader that a pre-trained potential computed at a "low" level of theory can be corrected using a small set of "high" level data. However, in my opinion. a

minor revision is still necessary since the following comments from the first reviewing report were not addressed appropriately.

We have made minor revisions in response to all comments.

Motivation: ——— The authors remain unclear about their central goal. In the response they state: "Very simply, our goal is to demonstrate that we can 'Delta-correct' to the CCSD(T) level for a transferable MLP of alkanes." In the manuscript they write: "We note here and also in a very recent paper that dispersion is not accounted for in these straight B3LYP energies. So the central objective of the work here is to correct this in a single step by using Delta-ML at the CCSD(T) level." These two claims are inconsistent and must be reconciled. Especially their claim that "However, the results in Figure 2 provide quantitative measure of dispersion over this large range of alkanes." should be carefully revised. The authors conflate method and basis-set errors with the physical dispersion term although this is for B3LYP mostly dispersion (but not exclusively). The authors should exchange their wording from the paper with the one provided in the response letter. We added a sentence stating that the Delta-ML approach deals with all deficiencies of DFT, i.e., dispersion, basis sets, exchange-correlation.

Citations: ——— The authors have not added enough citations, especially in the introduction (as previously commented). Specifically, the first two sentences of the introduction need additional and more appropriate citations. They read: "Hydrocarbons are important in fuels, plastics, and other industrial products. Consequently, there have been many studies, not only of their chemistry, but also of their physical and molecular properties." Citing Patterson alone is insufficient here. We added four new references. The abbreviation "aVDZ" should be introduced (aug-cc-pVDZ) and appropriate citations for both the functional and the basis set should be provided. We did this and added citations For the phrase "We note here and also in a very recent paper" a citation is missing. Please add it. Done.

Clarity: ———- As already mentioned in the first revision, sentences often wrongly ordered and the work is not self contained. Especially the following phrases are hard to follow and need the suggested rewrite: The sentence "We recently reported a transferable, many-body permutationally invariant polynomial (MB-PIP) potential for linear alkanes using roughly 253000 B3LYP electronic energies trained on C14 H30." needs rewriting because it reads as if B3LYP has been trained. I advise the authors to write the sentence like this: "We recently reported a transferable, many-body permutationally invariant polynomial (MB-PIP) potential for linear alkanes trained on roughly 253000 B3LYP/(insert basis set here) electronic energies of C14H30 conformations." Done. Also rewrite this sentence for clarity: "These focus on the minimum chain length where the transition from the linear minimum to the hairpin minimum occurs." I suggest: "These studies focus on identifying the shortest chain length at which the hairpin structure becomes more stable, i.e., energetically more favorable, than the linear structure." Done. For Formula (1) and (2) the authors must define every term. This has already been commented in the first revision. For example they should write that N refers to the number of atoms, V is a potential etc. This is currently not self contained. Otherwise they can mean anything and thus provides no worth to the manuscript. Done.

Configuration diversity: ———————— The authors have much improved by clarifying how the configurations for the coupled cluster calculations were obtained. However, still metrics for diversity have not been included in the revised manuscript. Figure 3 of the authors previous paper(https://nam11.safelinks.protection.outlook.com/?url=httpsDone.

Performance: ————— I agree with referee 1 that comparative benchmarks, particularly in terms of accuracy and computational efficiency are of high interest. The authors should remove their speculative statements from the paper and rather provide some performance and timing comparisons in the supporting information. We agree, but

that is largely beyond the scope of the current Letter. However, we have run timings of MACE-OFF and our MB-PIP potential for $C_{14}H_{30}$ and present those results near the end of the paper.

Additional Questions: Urgency: High

Significance: Moderate

Novelty: High

Scholarly Presentation: High
